# Data Management and Analysis of Metal–Organic Framework Synthesis Using Data Models

**DOI:** 10.1021/acs.jcim.6c00542

**Published:** 2026-05-08

**Authors:** Felix Neubauer, Kenichi Endo, Frederic Bender, Esengül Ciftci, Niels Hansen, Simon Krause, Benjamin Uekermann, Jürgen Pleiss

**Affiliations:** † Institute for Parallel and Distributed Systems, 9149 University of Stuttgart, Universitätsstraße 32, Stuttgart 70569, Germany; ‡ Institute of Polymer Chemistry, University of Stuttgart, Pfaffenwaldring 55, Stuttgart 70569, Germany; § Institute of Thermodynamics and Thermal Process Engineering, University of Stuttgart, Pfaffenwaldring 9, Stuttgart 70569, Germany; ∥ 28326 Max Planck Institute for Solid State Research, Nanochemistry Department, Heisenbergstraße 1, Stuttgart 70569, Germany; ⊥ Institute for Inorganic Chemistry II, Ulm University, Albert-Einstein-Allee 11, Ulm 89081, Germany; # Institute of Biochemistry, University of Stuttgart, Allmandring 31, Stuttgart 70569, Germany

## Abstract

The successful synthesis of metal–organic frameworks (MOFs) in high yield and purity critically depends on the details of the procedure. Therefore, the machine-readable as well as findable, accessible, interoperable, and reusable (FAIR) documentation of the synthesis procedure and the associated characterization data is crucial to ensure reproducibility and to enable data-driven analysis and systematic optimization of synthesis. Here, we demonstrate a data-processing workflow developed based on a JSON Schema data model for the synthesis and characterization of MOFs. Its feasibility and usefulness are demonstrated by synthesis data of two MOF systems, Fe–terephthalate MOF and MOCOF-1, and their subsequent characterization by powder X-ray diffraction (PXRD). The data model supports the development of an integrated workflow to (1) parse synthesis data from a table or an electronic lab notebook (ELN) into standardized JSON forms, (2) validate the data sets for errors and incompleteness, (3) serialize the data into the standardized data exchange formats MPIF and XDL, and (4) analyze PXRD data by a decision tree to identify critical synthesis parameters that control phase selectivity and yield. The data model and the workflow are modular and extensible and can be adapted to other data sources, characterizations, and AI methods for analysis. The proposed data model strategy makes MOF synthesis FAIR and AI-ready, fosters the digitalization of synthetic chemistry, and accelerates discovery.

## Graphical abstract

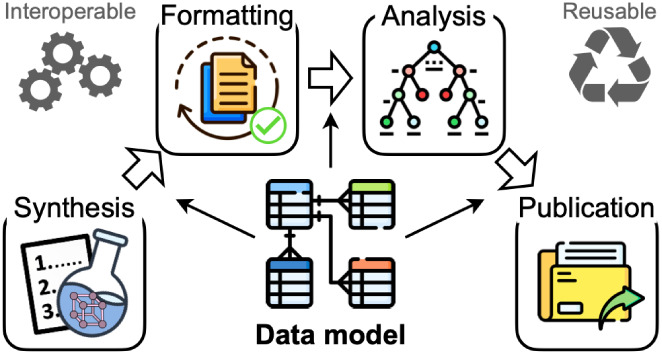

## Introduction

Metal–organic frameworks (MOFs) are highly designable porous materials with applications in gas storage, separation, sensing, and catalysis.^1–5^ Their formation is sensitive to various reaction parameters and is often difficult to rationally interpret and predict. Recent advances in data science, including artificial intelligence (AI) and machine learning (ML), have provided powerful tools to analyze the formation of MOFs in a data-driven manner to achieve synthesis condition prediction, optimization, and accelerated discovery.^6–18^ Such data-driven approaches are especially effective when a data set includes ″failed experiments″,^19–22^ underscoring the importance of rigorous data management besides publishing successful results. However, machine readability as well as integrity and standardization of data often pose a challenge in applying data-driven approaches.^23,24^ The synthetic procedures are usually recorded in text, whether physical notebooks or electronic lab notebooks (ELNs)^25^ are used. Such textual recording is not only poorly machine-readable but also nonstandardized and prone to erroneous, ambiguous, or incomplete descriptions. Most published articles and major databases of MOFs, such as the CSD MOF Collection^26^ and CoRE MOF,^27^ whose entries are linked to the original publications, provide only this type of textual data. While tables are often used as a more machine-readable format, they are still error-prone and limited to flat structures without hierarchies. These traditional data recording styles undermine the effectiveness of data-driven approaches, especially when data is subsequently reused, and sometimes also cause poor reproducibility of synthesis.^28^ Besides, nonstandardized data formats require the development of custom scripts specific to the data set, resulting in poorly reusable codes and unsustainable software development. These challenges become pronounced in large collaborative projects or when data or software is shared.

To address these issues, the FAIR (findable, accessible, interoperable, reusable) principles for data management^29–33^ and the FAIR4RS principles for research software management^34^ were proposed. In accordance with these principles, several hierarchical data formats and guidelines have been developed, such as the Chemical Description Language (XDL) by Cronin and coworkers for general chemical synthesis,^35^ the Material Preparation Information File (MPIF) for MOF synthesis by EU4MOFs,^36^ and others.^37–39^ While such standardized and structured formats are useful for data and software management, including analysis and reuse, their implementation in an actual research workflow of chemical synthesis requires significant effort and is therefore not widespread. As an alternative strategy, text data can be parsed using natural language processing (NLP),^15,33,40,41^ or more recently, large language models (LLMs),^11,38,42–44^ for text mining of large literature data. Although this approach can extract a few parameters such as linker molecules and solvent identity, the detailed synthetic protocols are difficult to parse, and the accuracy of parsing is questionable especially with LLMs.^36^ Therefore, the implementation of FAIR data formats in the workflow is key to accurate and efficient data-driven approaches.

In data science, data models are used to manage large data and foster machine readability, standardization, and data integrity. A data model describes the data format, structure, value types, required attributes, and other expected properties of the data. It can be directly used to validate data integrity while ensuring a machine-readable, standardized data structure. A data model also enables efficient and sustainable development of data management and analysis software by serving as a central blueprint in model-driven engineering (MDE), which systematically derives implementations and related codes from high-level models.^45^ There are several data models for chemistry, such as the EnzymeML data model for catalysis data^46^ and Allotrope Simple Model (ASM) for analytical data,^47^ but the use of data models and MDE in the comprehensive data management of chemical synthesis has yet to be demonstrated.

Here, we demonstrate the feasibility and usefulness of a data model for coherent management of MOF synthesis data, unifying formatting, validation, serialization, and analysis in a single workflow. We constructed a data model that captures synthesis procedures and powder X-ray diffraction (PXRD) measurements of MOFs and applied it to two data sets: (1) Fe–terephthalate MOF syntheses^48,91^ and (2) syntheses of MOCOF-1,^49^ including failed syntheses. Based on this model, we implemented a workflow that parses data from tables or ELN exports into a standardized hierarchical structure, validates it for errors and incompleteness, serializes it into XDL and MPIF for data exchange, and supports data-driven analysis to identify key synthetic parameters. This approach can be transferred to other material classes and areas of research as well.

## Results

### Data Model

The XDL format was previously developed for robotic automation of organic and nanomaterial synthesis, and covers most of the general synthetic operations in a step-by-step description.^50^ Based on the XDL format, we developed a data model in the JSON Schema language^51^ for a general formulation of MOF synthesis, using the open-source software MetaConfigurator.^52^ A schema was developed to define the synthetic information including reagents and steps such as mixing or heating (Figure 1, Figure S1, Table S2), and a second schema covered the product characterization by weighing and PXRD (Figure S2, Table S3). Its feasibility and usefulness are demonstrated by two use cases.

**Figure 1:**
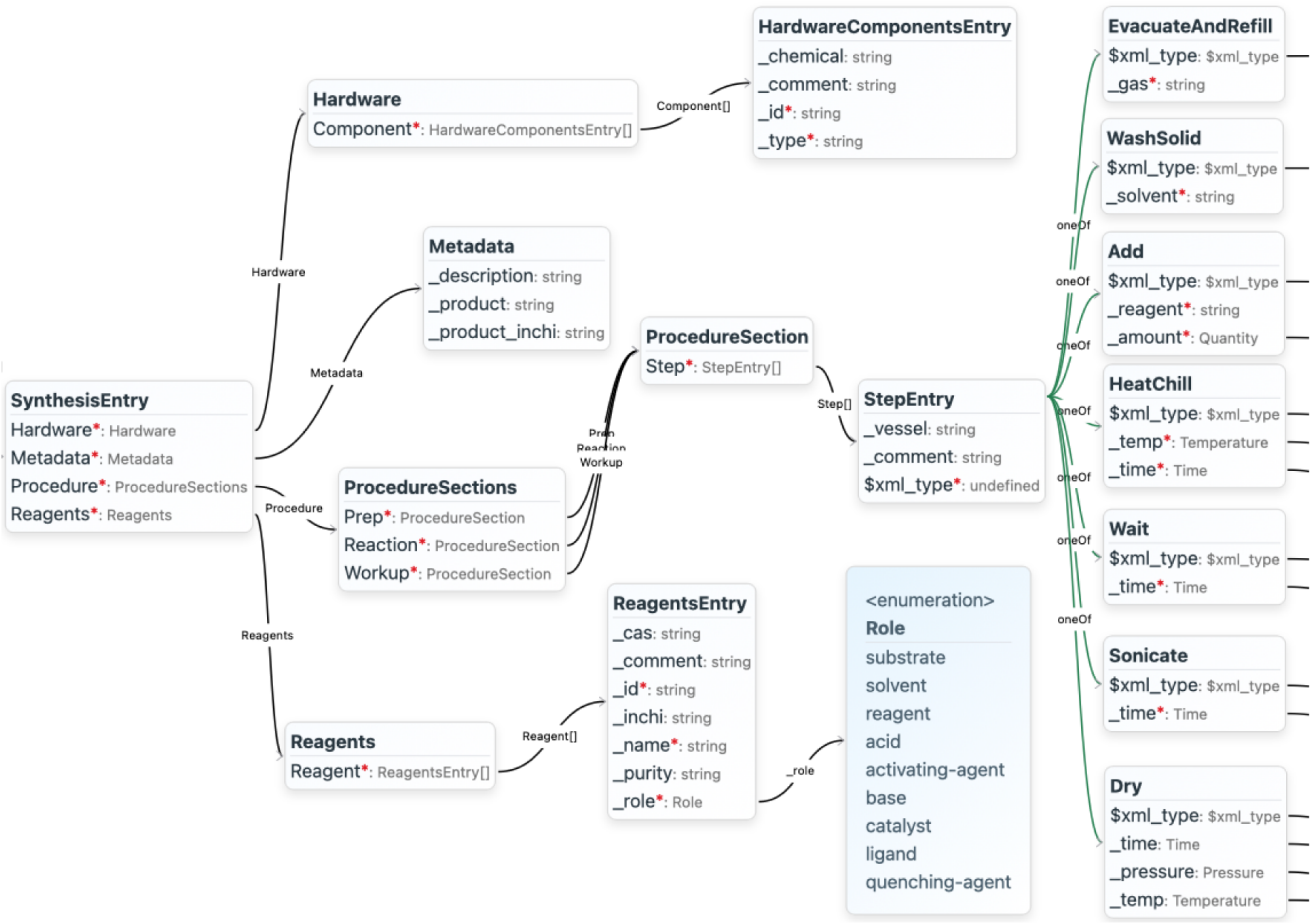
Graph representation of the data model for general MOF synthesis procedures, visualized by the software MetaConfigurator^52^ (truncated). The red asterisks denote the required properties. The full graph is given in Figure S1 and further descriptions for the schema classes are listed in Table S2.

#### Use Case 1: Data Formatting, Validation, and Serialization

The application of the MOF synthesis data model is demonstrated in a basic workflow for data formatting, validation, and serialization of a small data set on the synthesis of Fe–terephthalate (Fe–BDC) MOFs, which exhibits an exceptional multitude of phases despite its simple composition. From a single organic linker and Fe salt, four distinct frameworks can form: MIL-88B,^48^ MIL-101,^53,54^ MIL-53,^55,56^ and MIL-68 ^57,58^ (Figure 2a). The four phases span a wide range of mechanical properties, from flexible, breathing MIL-88B and MIL-53 to rigid MIL-101 and MIL-68. It is, thus, important to apply the appropriate synthesis conditions for obtaining the desired phase. However, the details of the synthetic methods are often incompletely described or omitted in the literature, limiting reproducibility.^48^ Seven synthesis experiments covering variations in metal salt, reagent amounts, modulator, and reaction temperature were conducted (Figure 2b, Table S1), and the products were characterized by PXRD (Figure S3). The synthesis conditions were recorded in a table together with information on the product phase determined by comparing PXRD patterns to the literature, representing the conventional style of recording MOF synthesis. The metadata for PXRD measurements were recorded implicitly by naming the respective files.

**Figure 2:**
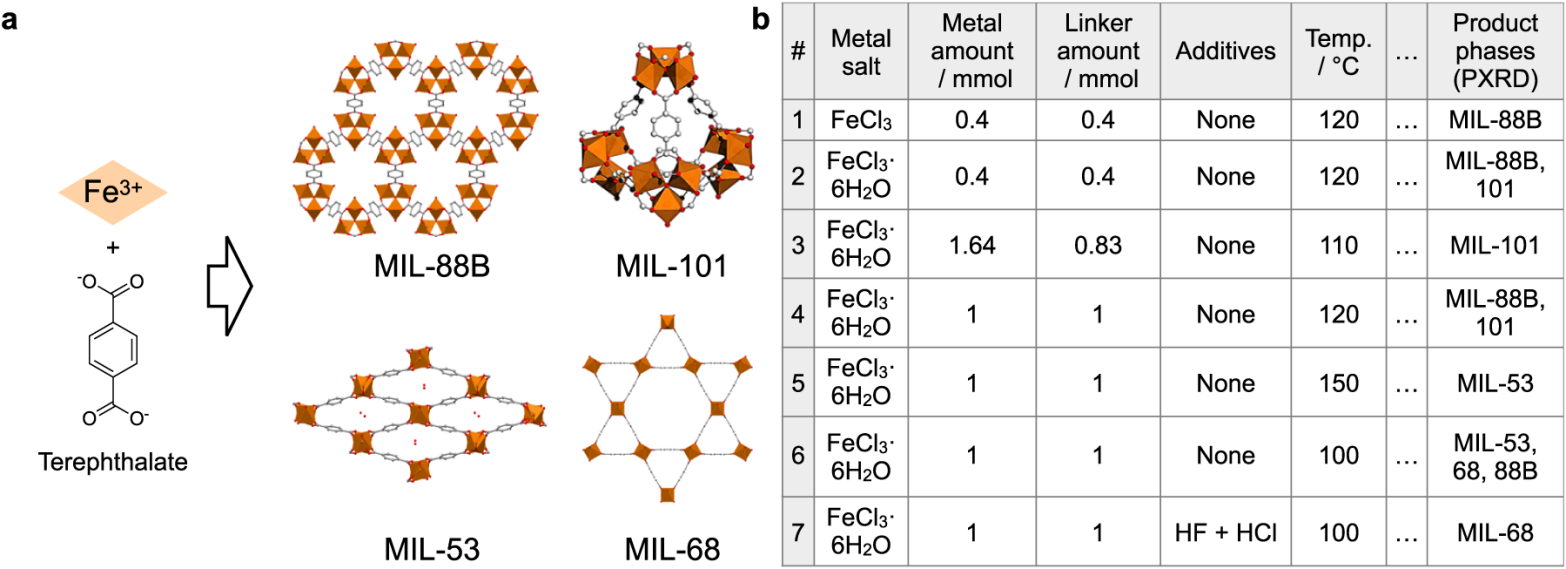
Data set of Fe–terephthalate MOF synthesis. (a) Reaction scheme and structures of product MOFs. (b) Table of synthetic conditions with selected columns. The full table is shown in Table S1.

In the workflow for formatting, validation, and serialization of MOF synthesis (Figure 3), each data operation was programmed in Python using data handling APIs generated from the data model with the quicktype library.^59^ We first used MetaConfigurator to convert the synthesis condition table from CSV into a JSON document, which was then parsed according to the data model. The PXRD data were converted into the X–Y data (XYD) format, and the PXRD metadata encoded in the filenames were also parsed into the data model. Data validation was achieved using the jsonschema Python library,^60^ which automatically checks for missing required properties or mismatch of value types with the data model. To demonstrate the reusability of this standardized data, we implemented data serialization into two existing formats, XDL and MPIF. Serialization into XDL was straightforward as the data model is based on XDL. Serialization into MPIF was realized using a TypeScript version of the data handling APIs to utilize the TypeScript library of MPIF.^61^

**Figure 3:**
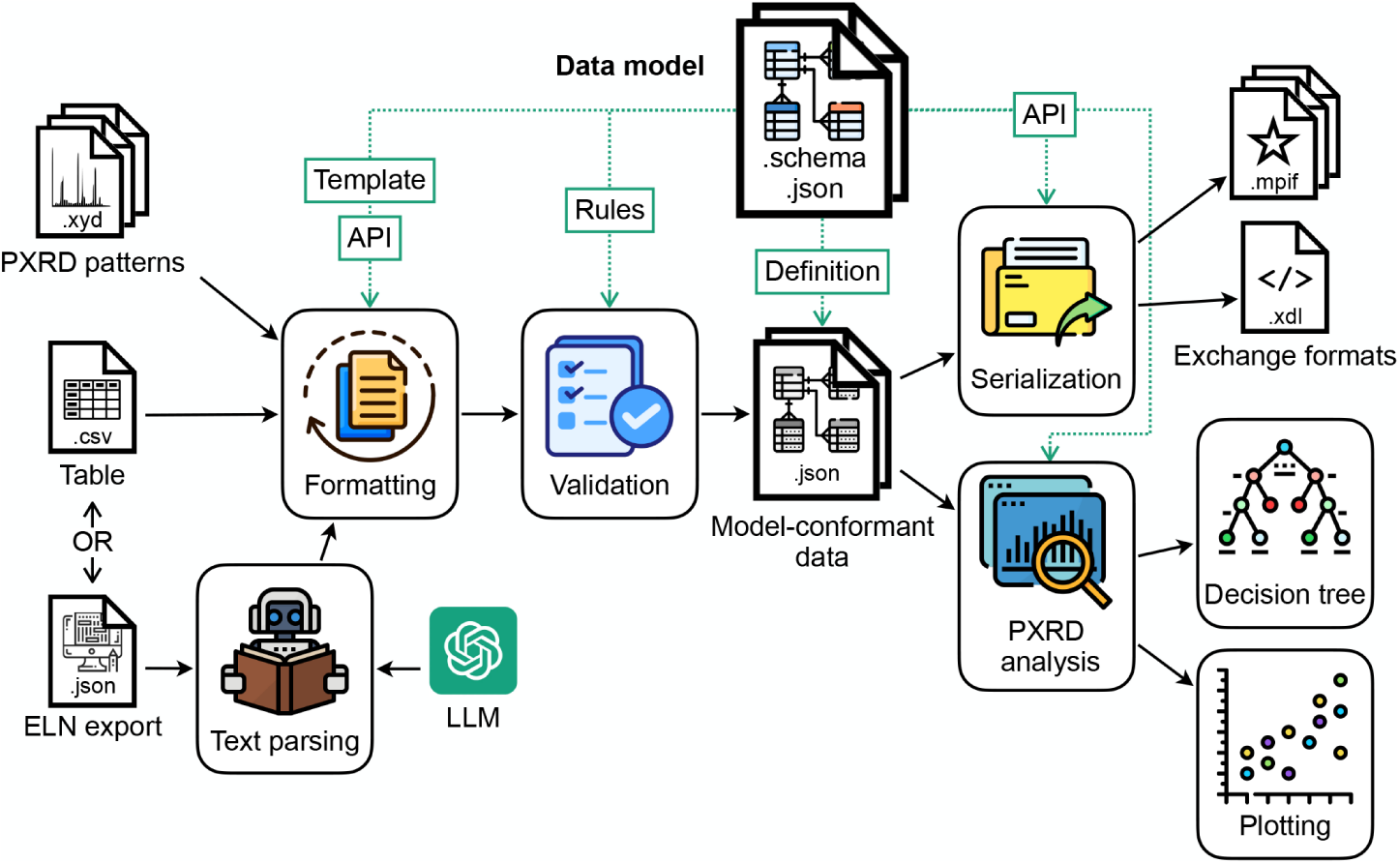
Workflow for the data processing of the MOF synthesis based on the data model. API, application programming interface; PXRD, powder X-ray diffraction; ELN, electronic lab notebook; LLM, large language model; .json, JavaScript Object Notation; .csv, comma-separated values; .xyd, X–Y data; .xdl, Chemical Description Language;^35^ .mpif, Material Preparation Information File.^36^

The overall workflow was able to automatically process the seven experimental entries of Fe–terephthalate MOF synthesis for formatting, validation, and serialization into XDL and MPIF. The validation of required properties mitigates the incomplete description of procedures, and the serialization into common formats facilitates data reuse, especially by the publication of the MOF synthesis results conforming to the MPIF standard. The obtained MPIF files were uploaded on the data repository DaRUS,^62^ conforming to the FAIR data publication (see Data Availability).

#### Use Case 2: ELN Integration, Text Parsing, and Data Analysis

As the second use case, an advanced workflow was developed that included the integration of an ELN, text parsing using an LLM, processing of PXRD data, and ML-based data analysis for linking synthesis conditions and MOF formation. The workflow was applied to a large data set of 183 samples originated from the synthesis of MOCOF-1, which was recently reported by Endo et al. as a special type of MOF incorporating features of covalent organic frameworks (COFs) (Figure 4a).^49^ This material is constructed via the simultaneous extension of Co–N coordination and imine condensation between 5,10,15,20-tetrakis­(4-aminophenyl)­porphinatocobalt­(II) (Co­(tapp)) and terephthalaldehyde (TPA), leading to an exceptional combination of crystallinity, porosity, and chemical stability. As its synthesis involves two different types of polymerization reactions, it is extremely sensitive to the reaction conditions. Suboptimal conditions led to side phases such as COF-366-Co (a COF with the same building blocks),^63,64^ amorphous coordination polymer [Co­(tapp)]_*n*_X_*m*_,^64^ or a solution of monomer Co (tapp), which lack either coordination or condensation linkages (Figure 4a, Figure S4).^49^ To achieve selective formation of MOCOF-1, 183 sets of experimental conditions were systematically tested and recorded on Sciformation ELN, including many ″failed″ experiments not reported in the previous paper. Therefore, this data set serves as a test case for evaluating the scalability, the integration of an ELN, and the training of a decision tree by positive and negative results.

**Figure 4:**
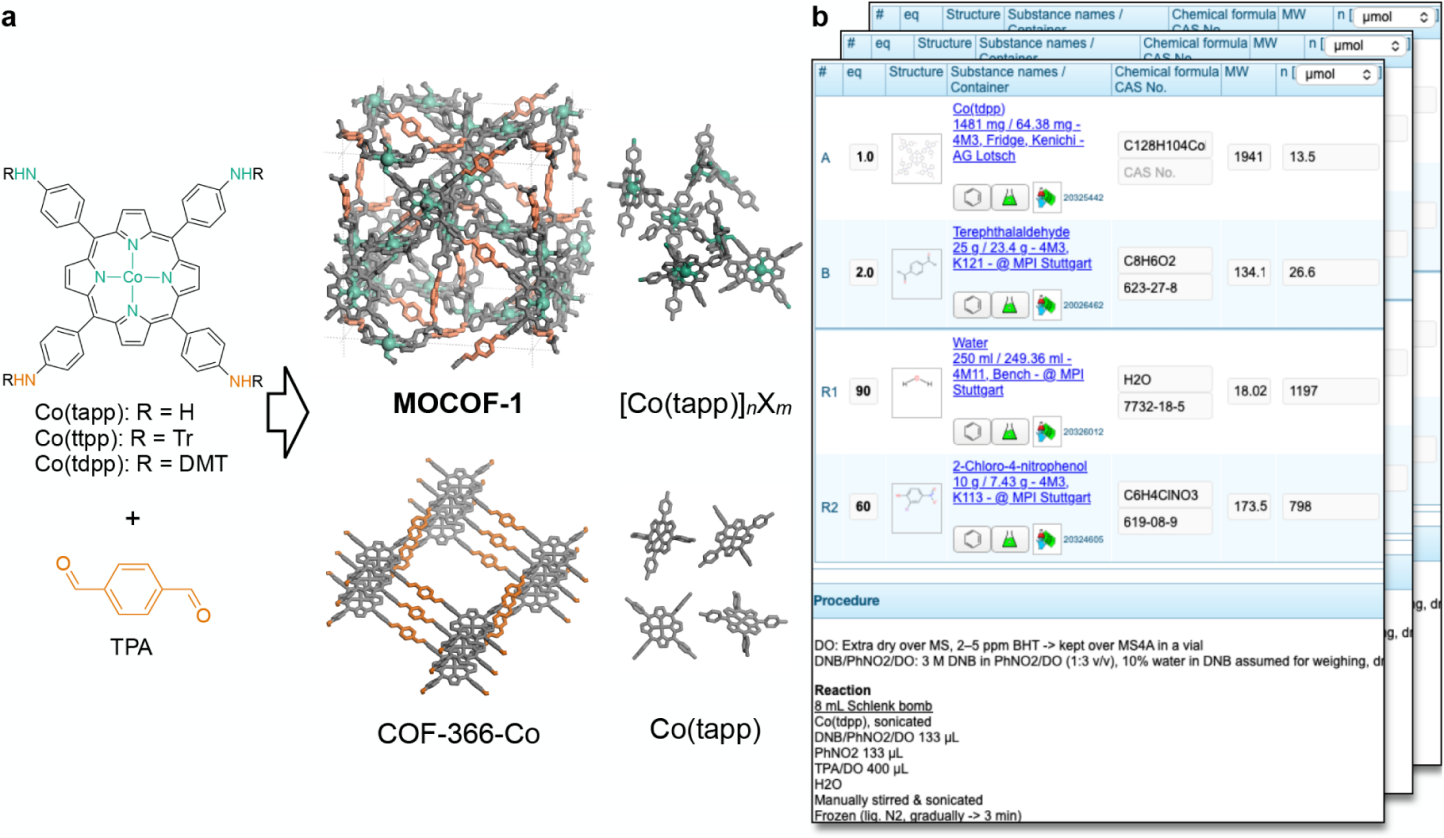
Data set of MOCOF-1 synthesis. (a) Synthetic scheme and structure of MOCOF-1 and the side phases. Tr, trityl (triphenylmethyl); DMT, 4,4’-dimethoxytrityl. Detailed chemical structures are shown in Figure S4. (b) Example synthesis entry on the electronic lab notebook Sciformation ELN, showing a table of chemicals and their amounts, as well as the initial part of the textual procedure description.

The synthetic procedures including a table of chemicals and their amounts as well as some textual description were recorded by a Sciformation ELN instrument (Figure 4b). The solid products were all characterized by PXRD to identify the product phases (Figure S5). Since PXRD cannot detect amorphous phases such as [Co­(tapp)]_*n*_X_*m*_, a few entries with single-phase formation were identified by combination with other characterization techniques such as N_2_ sorption and digestion ^1^H nuclear magnetic resonance (NMR), as detailed in the previous report.^49^ By comparison with these phase-pure samples, PXRD serves as a proxy measurement to roughly estimate the product purity. Solid product masses were also recorded on Sciformation ELN, which enables the approximate calculation of phase yields including the uncollected solution-phase product Co­(tapp).

A workflow to handle these data was developed based on the basic workflow of use case 1 (Figure 3). The data in Sciformation ELN was exported as a JSON instance. The textual description of the experimental procedures was parsed using the LLM gpt-4.1.-mini^65^ into properties defined by the data model, and mapped as described in use case 1. The accuracy of text parsing by the LLM was verified using a script with manually implemented rules. The LLM produced results that were identical to those of the rule-based script, in contrast to a previous report highlighting the unreliability of LLMs for extracting synthetic details.^36^ This high accuracy can be attributed to the use of the ELN that provides information on chemicals in a standardized JSON format, and to the fact that all of the textual descriptions were written consistently by a single author. For validation, the MOF synthesis schema was augmented by additional constraints relevant to this data set, such as regular expression patterns, minimal and maximal values, and enumerations (limited options) using the JSON Schema language. The modified schema allowed for precise validation of the data. The serialization of the formatted data into MPIF and XDL formats was achieved by the scripts described in use case 1. This workflow was able to generate 183 MPIF files from the ELN data by one command, showing scalable support for reusable data publication.

The workflow was further expanded to automatically analyze the characterization data, including PXRD patterns, to estimate product yields in two steps: first, the mole fractions of individual product phases were estimated from PXRD patterns by normalization, baseline subtraction, and linear-combination fitting with the phase-pure reference patterns. The reference patterns were selected by matching the retrieved metadata (the X-ray source and sample holder). Note that this quantification method provides only an approximation of phase mole fractions as the peak intensity of PXRD patterns can be affected by many factors. Second, the yield for each phase was calculated by combining the phase mole fractions with the amount of precursor and the product masses retrieved from the API.

Then, the relationships between the reaction conditions and the MOCOF-1 formation was analyzed by a decision tree model. To compensate for the model’s simplicity despite the large number of parameters, the main product phase was used as the target value instead of the individual phase yields, as done in previous applications of a decision tree model to MOF synthesis.^20,66^ The trained tree identified the critical parameters leading to the formation of the four different main products at the top three levels (Figure 5a). The first critical parameter was the Co­(tapp) precursor, where the use of the 4,4’-dimethoxytrityl-protected precursor Co­(tdpp) led to the preferential formation of MOCOF-1. This behavior is probably related to our previous finding that 4,4’-dimethoxytrityl cations produced by the deprotection of Co­(tdpp) slowly oxidizes Co^II^ in situ and thereby promotes the formation of MOCOF-1.^49^ When Co­(tdpp) is used, the decision tree indicates that a high amount of water per TPA is important to avoid the formation of COF-366-Co. This effect can be explained by the hydrolysis of imine links, thereby suppressing the excessive imine condensation. The p*K*_a_ of the acid reagent used was also found to be important to obtain MOCOF-1 over Co­(tapp), which presumably controls the equilibrium between 4,4’-dimethoxytrityl alcohol and 4,4’-dimethoxytrityl cation necessary for the aforementioned Co^II^ oxidation. The more important conditions can be identified by the full tree reaching completely separated classes (Figure S6).

**Figure 5:**
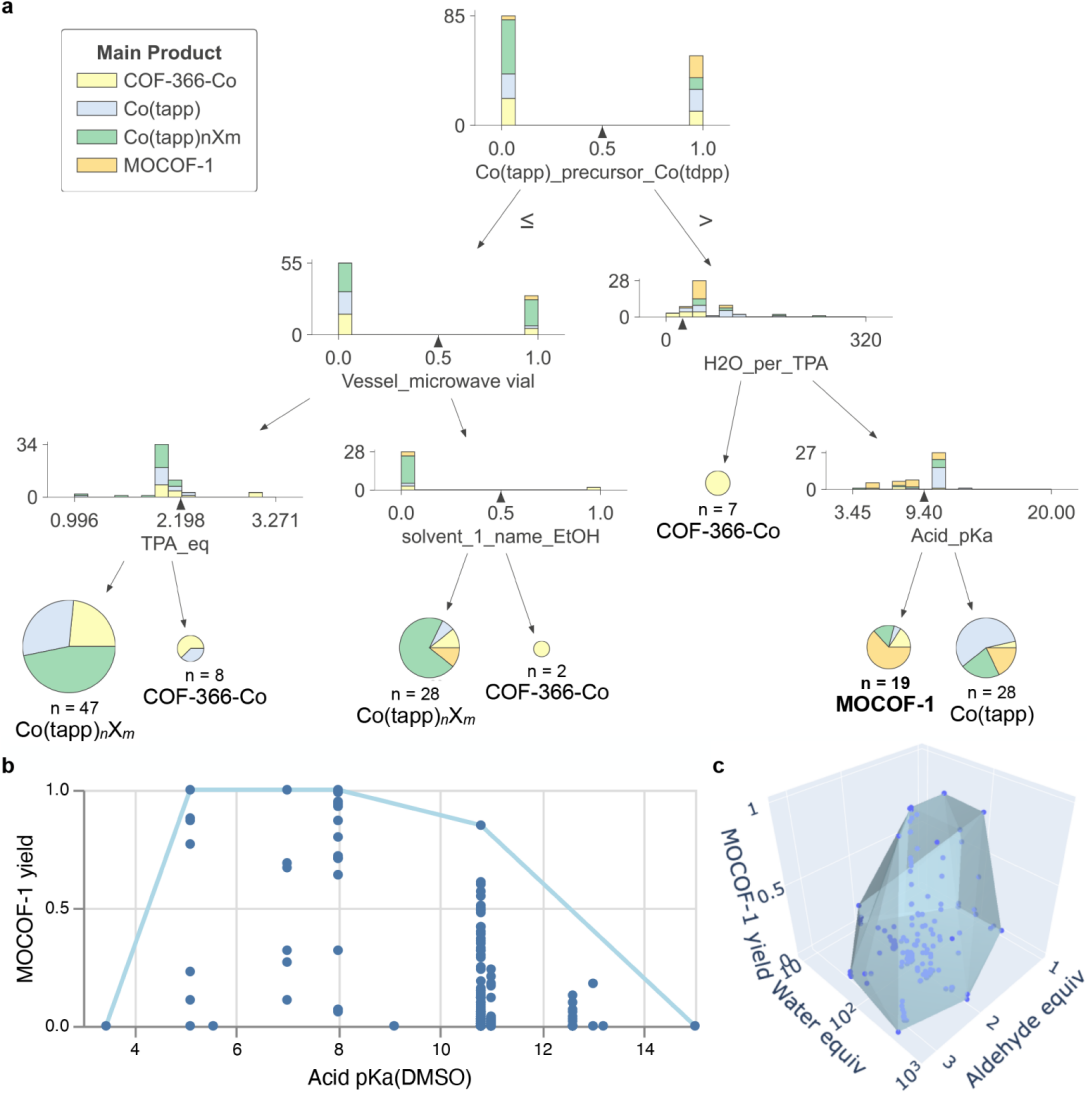
Data analysis results of the MOCOF-1 synthesis data set. (a) Decision tree visualizing the relationships between the reaction conditions and the main products. Each stacked bar plot indicates the sample statistics of a node and the splitting condition, while each pie chart denotes a terminal node with its statistics and main class. The tree was limited to three levels for this visualization; the tree for complete classification is given in Figure S6. (b) Convex hull plot of MOCOF-1 yield vs the p*K*_a_ of the used acid reagent in DMSO, visualizing their quantitative relationship under optimized conditions. (**c**) 3D convex hull plot of MOCOF-1 yield vs the equivalent amounts of the aldehyde and water to the Co–aminoporphyrin.

To further investigate the effects of the reaction conditions, we developed software to plot convex hulls. While the decision tree gave qualitative information on phase selectivity across many parameters, here we focus on obtaining quantitative information on MOCOF-1 yield regarding each parameter. As the decision tree identified the p*K*_a_ of the acid reagent to be critical for MOCOF-1 formation, we created a scatter plot of MOCOF-1 yield vs acid p*K*_a_. The plot showed a widespread of yield values on each p*K*_a_ value, reflecting the variation of many other parameters (Figure 5b). While the large number of parameters made it difficult to extract direct correlations, a convex hull enclosing all data points allowed visualization of the explored range of the parameter and the optimal results obtained so far. In this case, the quantitative formation of MOCOF-1 was achieved at the p*K*_a_ of 5–8 as long as other parameters are optimized. A similar convex hull was constructed in a 3D plot with TPA and water amounts, showing that optimized conditions are 1.5–2 equiv of the TPA and ca. 90 equiv of water (Figure 5c). These plots are interactive on the Marimo notebook platform^67^ where the plots can be rotated and the information on each data point can be revealed by mouseover.

The two use cases demonstrate the application of data processed using a data model to extract chemical insights. While we focused on identifying optimal conditions in the existing data set, this workflow can be extended to closed-loop optimization or chemical space exploration with more advanced ML tools. All experimental data, schemas, source codes, serialized data, and analysis results were made FAIR by publishing them on GitHub and the data repository DaRUS,^62^ together with a comprehensive metadata block (see Code and Data Availability).

## Discussion

Ensuring data integrity and standardization is increasingly recognized as a key challenge for using data-driven approaches^24^ and for achieving reproducibility^36^ in MOF synthesis. In this research, we developed a data model and a workflow for the data management and analysis of MOF synthesis. The data model is based on XDL^35^ and was applied in a workflow consisting of four steps: (1) parsing of data from a table or an ELN into standardized JSON instances for machine-readability; (2) data validation to exclude errors and incompleteness; (3) data serialization into the data exchange formats XDL and MPIF for standardized reporting; (4) data analysis to reveal the chemistry underlying MOF formation. In the case of MOCOF-1 synthesis, the last step elucidated important reaction parameters governing the formation of desired and side phases and their optimal value ranges (e.g., p*K*_a_ of the acid reagent being 5–8) to quantitatively obtain MOCOF-1.

The data model supported the code development for each step (Figure 3). (1) It served as a well-defined and structured template for the data. Its representation as a JSON Schema enabled efficient formatting with APIs and an LLM, reducing the manual effort commonly required when data formats are specified in nonstandardized documentation. (2) It provided rules for the rigorous validation of the formatted data to avoid data errors and incompleteness. The constraints defined in the JSON Schema were directly compared with the data using the jsonschema library,^60^ sparing manual implementation of rules. (3 and 4) The data model provided the semantics of the data structure for using the data, supported by APIs. It facilitated the serialization of the data into other formats and the complex analysis of the data because the relevant parameters could be easily retrieved. The same script was able to be used for serialization of two different MOF synthesis data sets by using the same data model. In addition, the explicit definition of templates, rules, and semantics by the data model streamlined the communication between chemists and software engineers during code development and enabled parallel development of the formatting, serialization, and analysis scripts, boosting the productivity of the team.

On a higher level, the data model supported the implementation of the FAIR^29^ and FAIR4RS^34^ principles for data and software. The standardization and definition of the data structure and properties, as well as their serialization, enhanced the findability, interoperability, and reusability of the data. Besides, data validation supported their reusability and reproducibility. The data model also defines the input of the software, which is important for sustainable software development and its reusability.

Because of their modularity, the workflow and the data model can be extended to describe the synthesis of other materials, to use additional characterization methods,^68^ and to further applications such as catalysis.^69^ The data source might also vary, such as other ELNs,^25,70^ high-throughput screening systems,^71^ robotic platforms,^10,14,72–75^ repositories, or literature if accurate text-mining is possible. The workflow is scalable and can handle large data sets. While we developed the data model by building on the existing standard XDL, suitable and problem-specific data models can be generated from any other standard, from in-house data formats, or from scratch if no suitable standard is available. To further conform with standards, text attributes might be linked to ontologies such as the Chemical Methods Ontology (CHMO) (e.g., by using the custom $iri keyword in the schema),^76,77^ and analytical methods might be represented using Allotrope Simple Models (ASM).^47^ The creation of data models from scratch does not require advanced data science knowledge when using MetaConfigurator, a user-friendly tool to generate schemas using natural language, interactive diagrams, and a GUI.^52,78^

Data models can be further utilized for developing workflows of chemical synthesis involving more advanced chemoinformatics tools and data science techniques,^7,24,79^ such as principal component analysis (PCA),^10^ random forest (RF),^10,15,19,20^ gradient boosting,^12^ or neural networks.^40,80^ The analysis results can be used to generate new sets of synthetic conditions for the closed-loop exploration of the chemical space guided by algorithms such as genetic algorithm,^14,19^ Bayesian optimization, or Gaussian process classification.^10,81^ The synthetic procedure, structured and validated using a data model, can also serve as an instruction to a synthesis robot^10,14,72–75^ or be used to build a database of synthetic protocols.

In conclusion, we presented that data models support the development of workflows for data management and analysis of MOF synthesis by enabling synthetic chemists to take advantage of the rapidly growing ecosystem of data science tools. Making chemical synthesis data FAIR and AI-ready will contribute to the cultural transformation of chemistry toward a data-driven discipline.

## Methods

### Experimental Procedures

No unexpected or unusually high safety hazards were encountered.

#### Synthesis Trials of Fe–Terephthalate MOFs

Seven synthesis entries (S-1 to S-7) were prepared under different conditions (Table S1). S-3 followed literature,^53,54^ while S-5 and S-7 were modifications from the literature.^55–58^ The reaction vessel was either a 12 mL glass vial or a 25 mL Teflon-lined autoclave.

#### Synthesis Trials of MOCOF-1

183 synthesis trials were conducted similarly to the reported procedure^49^ with variation of parameters (see Data Availability). An 8 mL Schlenk bomb or 7.5 mL microwave vial was used as the reaction vessel. Solvent was degassed by three freeze–pump–thaw cycles for some entries. Heating was conducted in a convection oven or an aluminum block on a hot plate. The solid products were collected by filtration and dried with supercritical CO_2_ or under vacuum.

#### PXRD Measurements

PXRD measurements were performed on a Stoe Stadi P diffractometer using Cu Kα_1_ or Co Kα_1_ radiation, monochromatized with a Ge(111) monochromator in Debye–Scherrer geometry at room temperature. Fe–terephthalate MOFs were analyzed directly after reaction in the mother liquor in ø 0.7 mm glass capillaries with sample rotation. Samples of synthetic trials for MOCOF-1 were measured after drying in ø 1.0 mm glass capillaries or as a ø 3 mm flat plate sealed between a pair of Kapton adhesive dots with sample rotation. The data files were named according to the experiment ID, the X-ray source, and the sample holder. The raw measurement data were converted by WinXPOW into the XYD (also known as XY) format.

### Data Modeling

The data model for MOF synthesis was implemented as two JSON schemas: the procedure schema for the synthetic procedures and the characterization schema for the product mass and PXRD measurements. These JSON schema were created using MetaConfigurator (commit 98160e1 ^82^), a versatile open-source web application equipped with a text editor, GUI editor, diagram editor, and AI-assistance.^52,78^ The procedure schema was created based on the documentation of XDL 2.0 Standard^50^ using the AI-assisted schema creation feature, with some customization for MOF synthesis (Figure S1). The characterization schema was created manually to include product mass, PXRD data relative file paths, and PXRD measurement metadata (Figure S2).

### Code Development and Data Processing

Scripts were written using Python (version 3.13). The Python APIs to use the schemas were generated by the library quicktype^59^ via MetaConfigurator.

#### Data Formatting and Validation

##### From Table

The condition table for the Fe–terephthalate MOF synthesis in the CSV format was imported into MetaConfigurator. From these data, a JSON schema was automatically inferred and then manually refined. The data was updated according to this refined schema using the AI-assisted mapping feature of MetaConfigurator^78^ and exported as a JSON instance. A script was implemented using the APIs generated from this schema and the procedure schema to format the JSON instance into a new JSON instance conforming to the procedure schema. This script was also programmed to search for the corresponding PXRD measurement data in the given directory and record its file path and the metadata from its filename in another JSON instance according to the characterization schema.

##### From Sciformation ELN

The data on Sciformation ELN for MOCOF-1 synthesis was exported as a JSON instance: The relevant experiment entries on Sciformation ELN were selected by its Search experiments command and then by using the Copy Query URL command. The obtained URL was edited by replacing startUseCase?useCase = performSearch & with performSearch? and appending &format = jsonRaw at the end, and the results were entered in a web browser to download a raw JSON instance containing the selected experimental entries. This raw JSON instance was preprocessed by Python scripts, which were developed to extract relevant parameters, convert the implicit notations (e.g., rxnRole 3 = solvent, the unit of amount = mol), and parse the textual description of procedures in realizationText using the LLM gpt-4.1 mini. The LLM was called via the OpenAI Python library^65^ with a prompt consisting of text-based parsing rules as a system message, the procedure text as a user message, and a JSON schema as a response format. The JSON schema was created and used here to control the response format, and then, the LLM response was further validated using this schema. The accuracy of parsing by the LLM was confirmed by running a separate script with manually implemented rules and comparing the outcomes. The preprocessed JSON instance was converted by another script into JSON instances conforming to the procedure and characterization schemas similarly to the Fe–terephthalate case.

##### Data Validation

At the end of the formatting scripts, the converted data was validated with the corresponding schema using the validate function of the jsonschema library (version 4.24.0).^60^ For the MOCOF-1 synthesis data, specific constraints were added to the procedure schema using MetaConfigurator before validation.

#### Data Serialization

##### Into XDL

A script was developed to convert the JSON format to the XML format. In this conversion, the array of operation steps were converted to their corresponding XDL tags using the helper attribute $xml_type to maintain the order of steps, while the Unit and Value was merged into one field using the helper attribute $xml_append.

##### Into MPIF

A third data file with additional metadata (e.g., author contact details) was created and used in addition to the synthesis procedure and characterization data. A TypeScript script was written utilizing the TypeScript APIs generated from the data model to convert these data into the MPIF standard using code from the MPIF Dashboard GitHub repository.^61^ The successful serialization was confirmed by uploading the file to the MPIF Dashboard.^83^

#### Data Analysis

General data handling was conducted with NumPy (version 2.2.4), SciPy (version 1.15.2), pandas (version 2.3.3), and polars (version 1.26.0) libraries. Experimental parameters important for analysis were extracted by a script from the procedure data via the APIs and saved as a JSON instance. Some parameters (e.g., p*K*_a_(DMSO) of acids) were added in this script according to the extracted parameters and the literature data.

##### Phase Mole Fraction Analysis of PXRD Patterns

The software was developed and used on a marimo web UI for notebook-style coding and interactive plotting.^67^ The PXRD data and metadata (X-ray source, sample holder) of the MOCOF-1 synthesis were retrieved using the data model API. The PXRD signal intensities depend on the irradiated sample volume, which can vary because of the variations in the packing efficiency, the capillary diameter for capillary measurements, and the sample amount for flat-plate measurements. To remove this effect, the signal intensities were normalized by the reference signal. For the Cu Kα_1_ measurement, the Co fluorescence signal at 2θ = 38–40° was used as reference, while for the Co Kα_1_ data surface scattering signal at 2θ = 1.5–1.8°. These reference signals are approximately proportional to the irradiated sample volume, and thus the normalized patterns can be used for phase quantification. Baselines were removed using the Statistics-Sensitive Nonlinear Iterative Peak Clipping (SNIP)^84–87^ algorithm, as implemented in the pybaselines (version 1.2.0) library.^88^ The settings were a maximum half-window of 40, a smoothing half-window of 3, and an enabled decreasing parameter. Mole fractions of each phase are estimated via linear combination fitting of reference patterns using non-negative least-squares (NNLS) optimization, implemented in SciPy. The reference patterns were chosen based on our previous paper,^49^ where the sample purity was confirmed by other characterization techniques. Suitable reference pattern for each measurement was selected by matching the X-ray source and the sample holder. The optimized NNLS weights corresponded to the mole fractions of each phase for the Cu Kα_1_ measurements, as the patterns were normalized by the Co fluorescence signal. The Co Kα_1_ measurements, normalized by the surface scattering signal, were assumed to approximate mole fractions. The amorphous fraction was estimated by the residual mole fractions, unassigned to crystalline phases. Note that this method only provides a rough approximation of phase mole fractions, as the PXRD signal intensities can be affected by various factors.

##### Phase Yield Calculation

Yields of individual product phases were calculated by combining the mole fractions with the precursor amounts and product masses retrieved via the APIs.

##### Decision Tree Modeling

Before training a decision tree, experiments with similar conditions (the difference of each parameter is less than 3% of the mean of the parameter) were removed to avoid skewing the training process. This process left 142 unique entries for training. Some parameters were combined or converted to minimize correlations between the parameters, e.g., the TPA amount and the Co–aminoporphyrin amount were combined into the TPA equivalent. Categorical parameters (e.g., solvent name) were encoded using One-Hot Encoding. Parameters with little relevance to the phase selectivity (e.g., workup parameters) were removed to lower the number of parameters. The resultant parameters were used as the features to train the decision tree using scikit-learn (version 1.3.2) setting the main product (phase with the highest mole fraction) as a target variable. The resulting decision tree was visualized with dtreeviz (version 2.2.2) for the detailed view of a small tree and with graphviz (version 0.21) for the overview of a large tree.

##### Convex Hull Plotting

Convex hull plots were created on a marimo web UI for interactive coding and plotting. Some parameters were converted in the same way as in the previous case to minimize correlations. Convex hulls were calculated using scipy.spatial. The 3D plots were created using plotly.express (version 6.3.1), while the 2D plots were created using altair (version 5.5.0).

## Supplementary Material



## Data Availability

All data supporting this study, including raw and processed data sets, are available in a public GitHub repository at: https://github.com/FAIRChemistry/mof-synthesis-data-modeling (commit 60a2682 ^90^) and in the data repository DaRUS at: 10.18419/DARUS-5695. The codes developed in this study are openly available in the same GitHub repository (https://github.com/FAIRChemistry/mof-synthesis-data-modeling, commit 60a2682 ^90^) under MIT License, together with documentation. MetaConfigurator is available in a separate GitHub repository (https://github.com/MetaConfigurator/meta-configurator), also under MIT License.

## Notes

Full table of experimental conditions for the synthesis of Fe–terephthalate MOFs, graph representations of the data model, PXRD patterns, the structure formulas of MOCOF-1 and side phases, and the decision tree for full classification (PDF)

## References

[1] Canossa S., Wuttke S., Barbour L. J. (2025). The Chemistry of Space: From Crystallographic
Abstraction to Framework Design. Angew. Chem.,
Int. Ed..

[2] Freund R. (2021). 25 Years of Reticular Chemistry. Angew. Chem.,
Int. Ed..

[3] Freund R., Zaremba O., Arnauts G., Ameloot R., Skorupskii G., Dinca M., Bavykina A., Gascon J., Ejsmont A., Goscianska J., Kalmutzki M., Lachelt U., Ploetz E., Diercks C. S., Wuttke S. (2021). The Current Status of MOF and COF
Applications. Angew. Chem., Int. Ed..

[4] Yaghi, O. M. ; Kalmutzki, M. J. ; Diercks, C. S. Introduction to Reticular Chemistry: Metal-Organic Frameworks and Covalent Organic Frameworks; John Wiley & Sons: Weinheim, 2019.

[5] Horike S., Kitagawa S. (2022). The Development of Molecule-Based Porous Material Families
and Their Future Prospects. Nat. Mater..

[6] Park J., Kim H., Kang Y., Lim Y., Kim J. (2024). From Data to Discovery:
Recent Trends of Machine Learning in Metal-Organic Frameworks. JACS Au.

[7] Lyu H., Ji Z., Wuttke S., Yaghi O. M. (2020). Digital Reticular Chemistry. Chem.

[8] Neikha K., Puzari A. (2024). Metal-Organic Frameworks through the Lens of Artificial
Intelligence: A Comprehensive Review. Langmuir.

[9] Ozcan A., Coudert F.-X., Rogge S. M. J., Heydenrych G., Fan D., Sarikas A. P., Keskin S., Maurin G., Froudakis G. E., Wuttke S., Erucar I. (2025). Artificial
Intelligence Paradigms
for Next-Generation Metal–Organic Framework Research. J. Am. Chem. Soc..

[10] Rong Z. (2026). Algorithmic Iterative Reticular Synthesis of Zeolitic Imidazolate
Framework Crystals. Nat. Synth..

[11] Kang Y., Lee W., Bae T., Han S., Jang H., Kim J. (2025). Harnessing
Large Language Models to Collect and Analyze Metal-Organic Framework
Property Data Set. J. Am. Chem. Soc..

[12] Pakamorė I., Forgan R. S. (2025). Computation-Guided
Exploration of the Reaction Parameter
Space of *N*, *N* -Dimethylformamide
Hydrolysis. Digital Discovery.

[13] Zheng Z., Zhang O., Nguyen H. L., Rampal N., Alawadhi A. H., Rong Z., Head-Gordon T., Borgs C., Chayes J. T., Yaghi O. M. (2023). ChatGPT Research
Group for Optimizing the Crystallinity
of MOFs and COFs. ACS Cent. Sci..

[14] Pilz L., Natzeck C., Wohlgemuth J., Scheuermann N., Weidler P. G., Wagner I., Wöll C., Tsotsalas M. (2023). Fully Automated Optimization of Robot-Based MOF Thin
Film Growth via Machine Learning Approaches. Adv. Mater. Interfaces.

[15] Luo Y., Bag S., Zaremba O., Cierpka A., Andreo J., Wuttke S., Friederich P., Tsotsalas M. (2022). MOF Synthesis Prediction Enabled
by Automatic Data Mining and Machine Learning. Angew. Chem., Int. Ed..

[16] Kitamura Y., Nakamura Y., Sugimoto K., Yoshikawa H., Tanaka D. (2022). Data-Driven Efficient Synthetic Exploration of Anionic
Lanthanide-Based Metal-Organic Frameworks. Chem.
Commun..

[17] Lee S., Jeong H., Jung S., Kim Y., Cho E., Nam J., ChangMo Yang D., Shin D. Y., Lee J.-H., Oh H., Choe W. (2025). Data-Driven
Search Algorithm for Discovery of Synthesizable Zeolitic
Imidazolate Frameworks. JACS Au.

[18] Livas C. G., Trikalitis P. N., Froudakis G. E. (2024). MOFSynth: A Computational Tool toward
Synthetic Likelihood Predictions of MOFs. J.
Chem. Inf. Model..

[19] Moosavi S. M., Chidambaram A., Talirz L., Haranczyk M., Stylianou K. C., Smit B. (2019). Capturing Chemical Intuition in Synthesis
of Metal-Organic Frameworks. Nat. Commun..

[20] Kitamura Y., Terado E., Zhang Z., Yoshikawa H., Inose T., Uji–i H., Tanimizu M., Inokuchi A., Kamakura Y., Tanaka D. (2021). Failure–Experiment–Supported
Optimization of Poorly Reproducible Synthetic Conditions for Novel
Lanthanide Metal–Organic Frameworks with Two–Dimensional
Secondary Building Units. Chem. - Eur. J..

[21] Raccuglia P., Elbert K. C., Adler P. D. F., Falk C., Wenny M. B., Mollo A., Zeller M., Friedler S. A., Schrier J., Norquist A. J. (2016). Machine-Learning-Assisted Materials
Discovery Using
Failed Experiments. Nature.

[22] Strieth-Kalthoff F., Sandfort F., Kühnemund M., Schäfer F. R., Kuchen H., Glorius F. (2022). Machine Learning for
Chemical Reactivity:
The Importance of Failed Experiments. Angew.
Chem., Int. Ed..

[23] Weber M. J., Guo Z., Zhang C., Schweidtmann A., Lapkin A. A. (2021). Chemical Data Intelligence
for Sustainable Chemistry. Chem. Soc. Rev..

[24] Chetry A. B., Ohto K. (2025). From Molecules to Data:
The Emerging Impact of Chemoinformatics in
Chemistry. J. Cheminf..

[25] Bird C. L., Willoughby C., Frey J. G. (2013). Laboratory Notebooks
in the Digital
Era: The Role of ELNs in Record Keeping for Chemistry and Other Sciences. Chem. Soc. Rev..

[26] Li A., Perez R. B., Wiggin S., Ward S. C., Wood P. A., Fairen-Jimenez D. (2021). The Launch of a Freely Accessible MOF CIF Collection
from the CSD. Matter.

[27] Zhao G. (2025). CoRE MOF DB: A Curated
Experimental Metal-Organic Framework Database
with Machine-Learned Properties for Integrated Material-Process Screening. Matter.

[28] Boström H. L.
B. (2024). How Reproducible
Is the Synthesis of Zr–Porphyrin
Metal–Organic Frameworks? An Interlaboratory Study. Adv. Mater..

[29] Wilkinson M. D. (2016). The FAIR Guiding Principles for Scientific Data Management and Stewardship. Sci. Data.

[30] Scheffler M., Aeschlimann M., Albrecht M., Bereau T., Bungartz H.-J., Felser C., Greiner M., Groß A., Koch C. T., Kremer K., Nagel W. E., Scheidgen M., Wöll C., Draxl C. (2022). FAIR Data Enabling New Horizons for
Materials Research. Nature.

[31] Stracke K., Evans J. D. (2024). The Rise of Data
Repositories in Materials Chemistry. Commun.
Chem..

[32] Pleiss J. (2024). FAIR Data
and Software: Improving Efficiency and Quality of Biocatalytic Science. ACS Catal..

[33] Wonanke, D. ; Longa, A. ; Pankajakshan, A. ; Himanen, L. ; Ladines, A. N. ; Márquez, J. A. ; Addicoat, M. A. ; Crittenden, D. ; Scheidgen, M. ; Lio, P. FAIR-MOFs: A Comprehensive Database for Accelerating the Discovery and Synthesis of Metal-Organic Frameworks ChemRxiv 2025 10.26434/chemrxiv-2025-zjjdc

[34] Barker M., Chue Hong N. P., Katz D. S., Lamprecht A.-L., Martinez-Ortiz C., Psomopoulos F., Harrow J., Castro L. J., Gruenpeter M., Martinez P. A., Honeyman T. (2022). Introducing the FAIR
Principles for Research Software. Sci. Data.

[35] Mehr S. H. M., Craven M., Leonov A. I., Keenan G., Cronin L. (2020). A Universal
System for Digitization and Automatic Execution of the Chemical Synthesis
Literature. Science.

[36] Cheung O., Tokuda S., Jędrzejowski D., Ploetz E., Baumgartner B., Pander M., Yang F., Evans J. D., Ettlinger R., Wuttke S., Matoga D. (2026). Material Preparation
Information
File (MPIF): A Community–Driven Standard for Reporting MOF
Syntheses. Adv. Mater..

[37] Smales, G. J. ; Appel, P. A. ; Breßler, I. ; Chambers, A. ; Dumele, O. ; Ebisch, M. ; Frontzek, J. ; Del Refugio Monroy, J. ; Rosalie, J. M. ; Pauw, B. R. DACHS and RoWaN: The Automated and Traceable Synthesis of ZIF-8 ChemRxiv 2025 10.26434/chemrxiv-2025-7fgg0

[38] Zhao, X. ; Langlois, K. ; Furst, J. ; McClellan, S. ; Hu, X. ; An, Y. ; Gómez-Gualdrón, D. A. ; Uribe-Romo, F. J. ; Greenberg, J. Metadata for Scientific Experiment Reporting: A Case Study in Metal-Organic Frameworks arXiv 2023 10.48550/arXiv.2310.12417

[39] Metal Organic Frameworks (MOFs) and Their Production Karlsruher Institut für Technologie; https://gitlab.kit.edu/kit/complat/metal-organic-frameworks/-/wikis/Home, (accessed 12/24/2025).

[40] Park H., Kang Y., Choe W., Kim J. (2022). Mining Insights on
Metal–Organic Framework Synthesis from Scientific Literature
Texts. J. Chem. Inf. Model..

[41] Glasby L. T., Gubsch K., Bence R., Oktavian R., Isoko K., Moosavi S. M., Cordiner J. L., Cole J. C., Moghadam P. Z. (2023). DigiMOF:
A Database of Metal-Organic Framework Synthesis Information Generated
via Text Mining. Chem. Mater..

[42] Zheng Z., Zhang O., Borgs C., Chayes J. T., Yaghi O. M. (2023). ChatGPT
Chemistry Assistant for Text Mining and the Prediction of MOF Synthesis. J. Am. Chem. Soc..

[43] Kalhor P., Jung N., Bräse S., Wöll C., Tsotsalas M., Friederich P. (2024). Functional Material Systems Enabled
by Automated Data Extraction and Machine Learning. Adv. Funct. Mater..

[44] Pruyn T. M., Aswad A., Khan S. T., Huang J., Black R., Moosavi S. M. (2025). MOF-ChemUnity: Literature-Informed Large Language Models
for Metal–Organic Framework Research. J. Am. Chem. Soc..

[45] Schmidt D. C. (2006). Guest Editor’s
Introduction: Model-driven Engineering. Computer.

[46] Range J., Halupczok C., Lohmann J., Swainston N., Kettner C., Bergmann F. T., Weidemann A., Wittig U., Schnell S., Pleiss J. (2022). EnzymeML—a
data
exchange format for biocatalysis and enzymology. FEBS J..

[47] Gardiner S., Haynie C., Della Corte D. (2024). Rise of the
Allotrope Simple Model:
Update from 2023 Fall Allotrope Connect. Drug
Discovery Today.

[48] Bara D., Meekel E. G., Pakamore I., Wilson C., Ling S., Forgan R. S. (2021). Exploring and Expanding the Fe-terephthalate Metal–Organic
Framework Phase Space by Coordination and Oxidation Modulation. Mater. Horiz..

[91] Ciftci E., Ortin-Rubio B., Tokuda S., Heck F., Stemmler F., Kuster K., Canossa S., Van Slageren J., Krause S. (2026). A Toolkit for Phase Identification and Reproducible
Synthesis of Fe-Terephthalate Metal-Organic Frameworks: MIL-88B, MIL-101,
MIL-53, and MIL-68. ChemRxiv.

[49] Endo K., Canossa S., Heck F., Proserpio D. M., Istek M. S., Stemmler F., van Slageren J., Hartmann S., Hartschuh A., Lotsch B. V. (2025). Crystalline Porous
Frameworks Based on Double Extension of Metal–Organic and Covalent
Organic Linkages. Nat. Synth..

[50] XDL 2.0 Standard Cronin Group; https://croningroup.gitlab.io/chemputer/xdl/standard/index.html, (accessed 12/19/2025).

[51] Pezoa, F. ; Reutter, J. L. ; Suarez, F. ; Ugarte, M. ; Vrgoč, D. Foundations of JSON Schema. In Proceedings of the 25th International Conference on World Wide Web; ACM, 2016, pp. 263–273. 10.1145/2872427.2883029.

[52] Neubauer F., Bredl P., Xu M., Patel K., Pleiss J., Uekermann B. (2024). MetaConfigurator: A User-Friendly
Tool for Editing
Structured Data Files. Datenbank–Spektrum.

[53] Férey G., Mellot-Draznieks C., Serre C., Millange F., Dutour J., Surblé S., Margiolaki I. (2005). A Chromium Terephthalate-Based Solid
with Unusually Large Pore Volumes and Surface Area. Science.

[54] Li Z., Liu X., Jin W., Hu Q., Zhao Y. (2019). Adsorption Behavior
of Arsenicals on MIL-101­(Fe): The Role of Arsenic Chemical Structures. J. Colloid Interface Sci..

[55] Millange F., Guillou N., Walton R. I., Grenèche J.-M., Margiolaki I., Férey G. (2008). Effect of
the Nature of the Metal
on the Breathing Steps in MOFs with Dynamic Frameworks. Chem. Commun..

[56] Pu M., Guan Z., Ma Y., Wan J., Wang Y., Brusseau M. L., Chi H. (2018). Synthesis of Iron-Based
Metal-Organic
Framework MIL-53 as an Efficient Catalyst to Activate Persulfate for
the Degradation of Orange G in Aqueous Solution. Appl. Catal., A.

[57] Liu Z., Li Q., Zhu H., Lin K., Deng J., Chen J., Xing X. (2018). 3D Negative Thermal Expansion in
Orthorhombic MIL-68 (In). Chem. Commun..

[58] Fateeva A., Horcajada P., Devic T., Serre C., Marrot J., Grenèche J.-M., Morcrette M., Tarascon J.-M., Maurin G., Férey G. (2010). Synthesis,
Structure, Characterization, and Redox Properties
of the Porous MIL–68­(Fe) Solid. Eur.
J. Inorg. Chem..

[59] Glideapps/Quicktype Glide; https://github.com/glideapps/quicktype, (accessed 11/12/2025).

[60] Jsonschema PyPi; https://pypi.org/project/jsonschema/. (accessed 01/05/2026).

[61] Yang, F. MPIF-GUI GitHub; https://github.com/fengxuyy/MPIF-GUI (accessed 12/24/2025).

[62] DaRUS University of Stuttgart; https://www.izus.uni-stuttgart.de/en/fokus/darus/. (accessed 01/29/2026).

[63] Lin S., Diercks C. S., Zhang Y. B., Kornienko N., Nichols E. M., Zhao Y., Paris A. R., Kim D., Yang P., Yaghi O. M., Chang C. J. (2015). Covalent Organic
Frameworks Comprising Cobalt Porphyrins for Catalytic CO2 Reduction
in Water. Science.

[64] Endo K., Raza A., Yao L., Van Gele S., Rodriguez-Camargo A., Vignolo-Gonzalez H. A., Grunenberg L., Lotsch B. V. (2024). Downsizing Porphyrin
Covalent Organic Framework Particles Using Protected Precursors for
Electrocatalytic CO(2) Reduction. Adv. Mater..

[65] Openai-Python OpenAI; https://github.com/openai/openai-python. (accessed 11/12/2025).

[66] Wakiya T., Kamakura Y., Shibahara H., Ogasawara K., Saeki A., Nishikubo R., Inokuchi A., Yoshikawa H., Tanaka D. (2021). Machine-Learning-Assisted
Selective Synthesis of a
Semiconductive Silver Thiolate Coordination Polymer with Segregated
Paths for Holes and Electrons. Angew. Chem.,
Int. Ed..

[67] Agrawal, A. ; Scolnick, M. Marimo - an Open-Source Reactive Notebook for Python; https://github.com/marimo-team/marimo. (accessed 12/24/2025).

[68] Huang, Y.-C. ; Tremouilhac, P. ; Kuhn, S. ; Huang, P.-C. ; Lin, C.-L. ; Schlörer, N. ; Taubert, O. ; Götz, M. ; Jung, N. ; Bräse, S. (Semi-) Automatic Review Process for Common Compound Characterization Data in Organic Synthesis ChemRxiv 2025 10.26434/chemrxiv-2024-1r9tb-v2

[69] Takahashi K., Ohyama J., Nishimura S., Fujima J., Takahashi L., Uno T., Taniike T. (2023). Catalysts
Informatics: Paradigm Shift towards Data-Driven
Catalyst Design. Chem. Commun..

[70] Tristram F., Jung N., Hodapp P., Schröder R. R., Wöll C., Bräse S. (2024). The Impact
of Digitalized Data Management
on Materials Systems Workflows. Adv. Funct.
Mater..

[71] Caldentey X., Romero E. (2023). High-Throughput Experimentation
as an Accessible Technology
for Academic Organic Chemists in Europe and Beyond. Chem.: Methods.

[72] Jiang Z., Liu Y., Ke Z. (2025). Recent Progress in Autonomous Laboratories for Chemical
Synthesis. Commun. Comput. Chem..

[73] Rauschen R., Ayme J.-F., Matysiak B. M., Thomas D., Cronin L. (2025). A Programmable
Modular Robot for the Synthesis of Molecular Machines. Chem.

[74] Dai T., Vijayakrishnan S., Szczypinski F. T., Ayme J. F., Simaei E., Fellowes T., Clowes R., Kotopanov L., Shields C. E., Zhou Z., Ward J. W., Cooper A. I. (2024). Autonomous
Mobile Robots for Exploratory Synthetic Chemistry. Nature.

[75] Basford A. R., Bernardino A. H., Teeuwen P. C. P., Egleston B. D., Humphreys J., Jelfs K. E., Nitschke J. R., Riddell I. A., Greenaway R. L. (2025). Development
of an Automated Workflow for Screening the Assembly and Host–Guest
Behavior of Metal–Organic Cages Towards Accelerated Discovery. Angew. Chem., Int. Ed..

[76] Pachl, C. ; Frank, N. ; Breitbart, J. ; Bräse, S. Overview of Chemical Ontologies arXiv 2020 10.48550/ARXIV.2002.03842

[77] Rsc-Cmo rsc-ontology; https://github.com/rsc-ontology/rsc-cmo, (accessed 11/12/2025).

[78] Neubauer, F. ; Uekermann, B. ; Pleiss, J. AI-assisted JSON Schema Creation and Mapping. In 2025 ACM/IEEE 28th International Conference on Model Driven Engineering Languages and Systems Companion (MODELS-C); IEEE, 2025, pp. 79–83. DOI: 10.48550/arXiv.2508.05192 10.1109/MODELS-C68889.2025.00019.

[79] Yano J., Gaffney K. J., Gregoire J., Hung L., Ourmazd A., Schrier J., Sethian J. A., Toma F. M. (2022). The Case for Data
Science in Experimental Chemistry: Examples and Recommendations. Nat. Rev. Chem..

[80] Feng B., Wang B., Lv L., Zhang M., Chen Z., Pan F., Li S. (2026). Interpreting
X-ray Diffraction Patterns of Metal–Organic
Frameworks via Generative Artificial Intelligence. J. Am. Chem. Soc..

[81] Gromski P. S., Henson A. B., Granda J. M., Cronin L. (2019). How to Explore Chemical
Space Using Algorithms and Automation. Nat.
Rev. Chem..

[82] MetaConfigurator Commit 98160e1 GitHub; https://github.com/MetaConfigurator/meta-configurator/tree/98160e1265d95af1435e04f4f0dc511049e533ca, (accessed 01/21/2026).

[83] MPIF Dashboard; http://mpif.jackdevans.com/, (accessed 11/12/2025).

[84] Ryan C., Clayton E., Griffin W., Sie S., Cousens D. (1988). SNIP, a Statistics-Sensitive
Background Treatment for the Quantitative Analysis of PIXE Spectra
in Geoscience Applications. Nucl. Instrum. Methods
Phys. Res., Sect. B.

[85] Morháč M., Kliman J., Matoušek V., Veselskỳ M., Turzo I. (1997). Background Elimination Methods for
Multidimensional Coincidence *γ*-Ray Spectra. Nucl. Instrum.
Methods Phys. Res., Sect. A.

[86] Morháč M., Matoušek V. (2008). Peak Clipping Algorithms for Background
Estimation
in Spectroscopic Data. Appl. Spectrosc..

[87] Morháč M. (2009). An Algorithm
for Determination of Peak Regions and Baseline Elimination in Spectroscopic
Data. Nucl. Instrum. Methods Phys. Res., Sect.
A.

[88] Erb, D. Pybaselines: A Python Library of Algorithms for the Baseline Correction of Experimental Data GitHub; https://github.com/derb12/pybaselines. (accessed 11/12/2025).

[89] Brand A., Allen L., Altman M., Hlava M., Scott J. (2015). Beyond authorship:
Attribution, contribution, collaboration, and credit. Learned Publ..

[90] Repository for Data management and analysis of metal–organic framework synthesis using data models, Commit 60a2682; https://github.com/FAIRChemistry/mof-synthesis-data-modeling/tree/60a2682f224cea4965bd7f4b7c989ce2ad7b1812. (accessed 04/15/2026). 10.1021/acs.jcim.6c00542 PMC13213836 42102309

