# Supplementary material for: Data Management and Analysis of Metal–Organic Framework Synthesis Using Data Models
Source: J Chem Inf Model. 2026 May 8;66(10):5869–79. doi: 10.1021/acs.jcim.6c00542 (PMC13213836; doi:10.1021/acs.jcim.6c00542)
Supplement: Supplementary file 1 [file ci6c00542_si_001.pdf]

Supporting information

Data management and analysis of metal–organic framework synthesis using data models

Felix Neubauer[†1], Kenichi Endo[†*2], Frederic Bender[3], Esengül Ciftci[4,5], Niels Hansen[3], Simon Krause[4,5], Benjamin Uekermann[1], and Jürgen Pleiss[*6]

[1] Institute for Parallel and Distributed Systems, University of Stuttgart, Universitätsstraße 32, 70569 Stuttgart, Germany

[2] Institute of Polymer Chemistry, University of Stuttgart, Pfaffenwaldring 55, 70569 Stuttgart, Germany

[3] Institute of Thermodynamics and Thermal Process Engineering, University of Stuttgart, Pfaffenwaldring 9, 70569 Stuttgart, Germany

[4] Max Planck Institute for Solid State Research, Nanochemistry Department, Heisenbergstraße 1, 70569 Stuttgart, Germany

[5] Institute for Inorganic Chemistry II, Ulm University, Albert-Einstein-Allee 11, 89081 Ulm, Germany

[6] Institute of Biochemistry, University of Stuttgart, Allmandring 31, 70569 Stuttgart, Germany

[†]: Authors contributed equally

* Corresponding authors:

Kenichi Endo, kenichi.endo@ipoc.uni-stuttgart.de

Jürgen Pleiss, juergen.pleiss@itb.uni-stuttgart.de

Supporting tables

Table S1. Table of the reaction conditions and workup procedures for Fe–terephthalate MOFs.

Vial No	S-1	S-2	S-3	S-4	S-5	S-6	S-7
**Date**	10.10.2025	10.10.2025	10.10.2025	10.10.2025	10.10.2025	10.10.2025	10.10.2025
**Metal salt name**	$FeCl_3$	$FeCl_3{\cdot}6H_2O$	$FeCl_3{\cdot}6H_2O$	$FeCl_3{\cdot}6H_2O$	$FeCl_3{\cdot}6H_2O$	$FeCl_3{\cdot}6H_2O$	$FeCl_3{\cdot}6H_2O$
**Metal salt mass**	0.4	0.4	1.64	1	1	1	1
**Metal salt mass unit**	mmol	mmol	mmol	mmol	mmol	mmol	mmol
**Linker name**	Benzene-1,4-dicarboxylic acid	Benzene-1,4-dicarboxylic acid	Benzene-1,4-dicarboxylic acid	Benzene-1,4-dicarboxylic acid	Benzene-1,4-dicarboxylic acid	Benzene-1,4-dicarboxylic acid	Benzene-1,4-dicarboxylic acid
**Linker mass**	0.4	0.4	0.83	1	1	1	2
**Linker mass unit**	mmol	mmol	mmol	mmol	mmol	mmol	mmol
**Solvent**	DMF	DMF	DMF	DMF	DMF	DMF	DMF
**Solvent unit**	ml	ml	ml	ml	ml	ml	ml
**Solvent amaount**	4	4	10	5	5	10	10
**Modulator**	no	no	no	no	no	no	5M HF, 1M HCl
**Modulator unit**	µl	µl	µl	µl	µl	µl	µl
**Modulator amount**	no	no	no	no	no	no	120+120
**Sonicator time**	30 min	30 min	30 min	30 min	30 min	30 min	30 min
**Reaction vessel**	glass vial	glass vial	Teflon lined autoclave	glass vial	Teflon lined autoclave	Teflon lined autoclave	Teflon lined autoclave
**Place**	oven	oven	oven	oven	oven	oven	oven
**Temperature**	120	120	110	120	150	100	100
**Temperature Unit**	deg C	deg C	deg C	deg C	deg C	deg C	deg C
**Reaction Time**	1	1	1	3	3	7	5
**Reaction Time Unit**	day	day	day	day	day	day	day
**Washing solids**	with Ethanol	with Ethanol	with Ethanol	with Ethanol	with Ethanol	with Ethanol	with Ethanol
**Drying solids**	under vacuum	under vacuum	under vacuum	under vacuum	under vacuum	under vacuum	under vacuum
**Activation temperature**	120	120	120	120	120	120	120
**Activation Temperature Unit**	deg C	deg C	deg C	deg C	deg C	deg C	deg C
**Drying solids time**	1	1	1	1	1	1	1
**Drying Time Unit**	day	day	day	day	day	day	day
**Phase Purity**	yes	no	yes	no	yes	no	yes
**Product MOF**	**MIL-88B (Fe)**	**MIL-88B+MIL101 (mix phases)**	**MIL-101 (Fe)**	**MIL-88B+MIL101 (mix phases)**	**MIL-53 (Fe)**	**MIL-53 (Fe)+MIL-68 (Fe)+MIL88B (Fe)**	**MIL-68 (Fe)**

Table S2. Schema classes defined in the MOF synthesis procedure schema (procedure.schema.json).

**Schema Class**	**Description**
Synthesis Procedure	An array of the recorded synthesis trials.
SynthesisEntry	A recorded synthesis trial.
Hardware	An array of the used reaction vessels.
HardwareComponentsEntry	A used reaction vessel.
Metadata	Metadata of the synthesis trial.
Reagents	An array of the used chemicals.
ReagentsEntry	A used chemical.
ProcedureSections	The whole synthesis procedure, divided into the preparation, reaction, and workup sections.
ProcedureSection	An array of the procedure steps in each section.
StepEntry	An individual procedure step, which is one of the following types.
EvacuateAndRefill	Evacuate the system and refill it with the given gas.
WashSolid	Rinse the solid products with the given solvent upon filtration.
Add	Add the given chemical to the reaction mixture.
HeatChill	Heat or chill the reaction mixture at the given temperature for the given time.
Wait	Wait for the given time.
Sonicate	Sonicate the reaction mixture for the given time.
Dry	Dry the solid products for the given time at the given vacuum pressure and the given temperature.
Amount	The amount of a reagent added to the vessel during a step.
Temperature	The heat/chill temperature during a step.
Time	The time duration of a step.
Pressure	The pressure during a step.

Table S3. Schema classes defined in the MOF characterization schema (characterization.schema.json).

**Schema Class**	**Description**
Characterization List	An array of the recorded characterization datasets.
CharacterizationEntry	A characterization dataset of the synthesis trial specified with ExperimentId.
Characterization	A set of characterization data containing PXRD and weight measurements.
Weight	A data from weighing the solid products.
PxrdMetadata	The data and metadata of a PXRD measurement.
SampleHolder	The sample holder used in the PXRD measurement.

Supporting figures

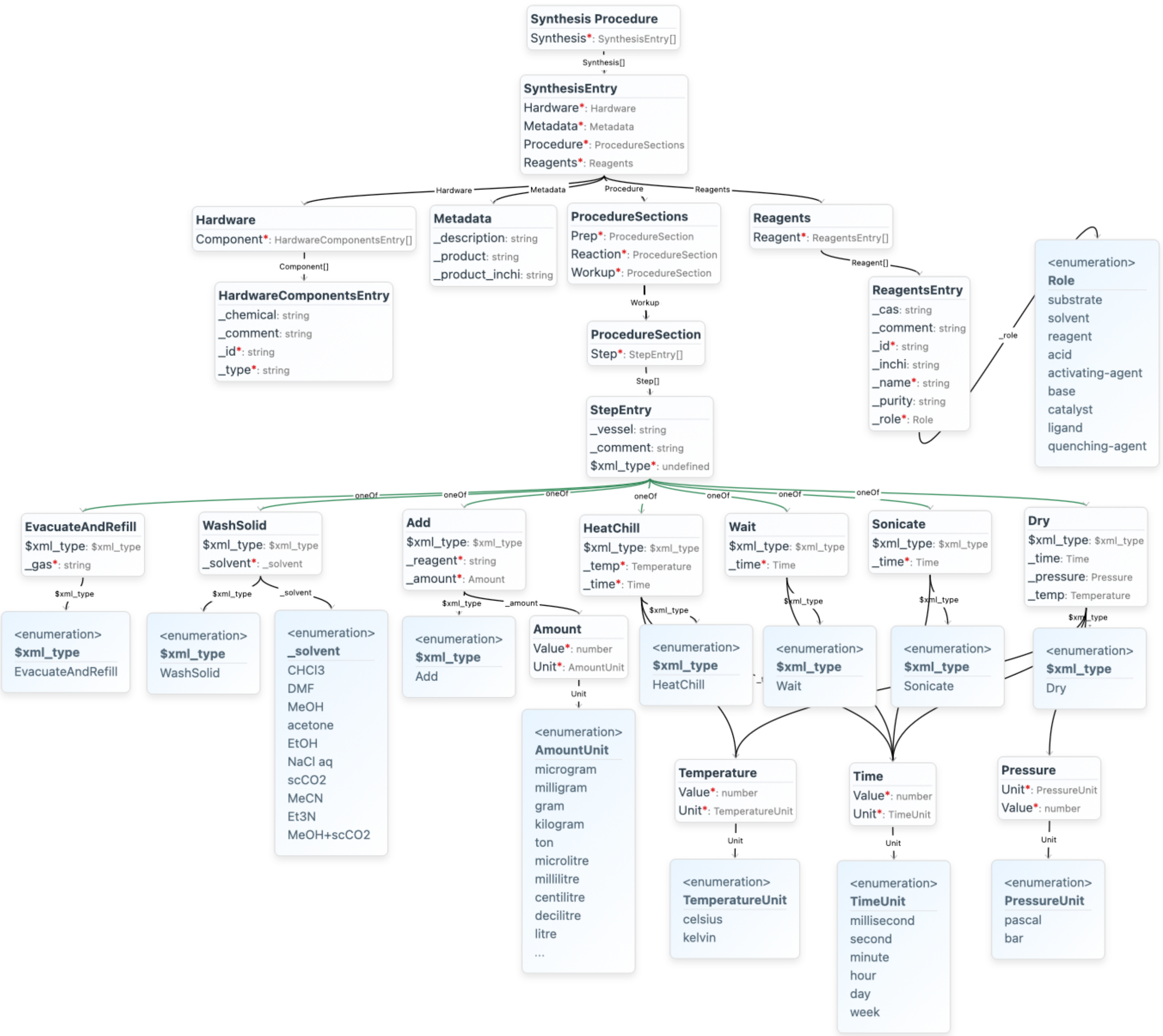

Figure S1. Graph representation of the data model for general MOF synthesis, drawn by the software MetaConfigurator. Red asterisks denote required properties. The classes are described in more detail in Table S2. The attribute *$xml_type* is used for the XDL-serialization. Boxes with an enumeration tag denote sets of permitted values.

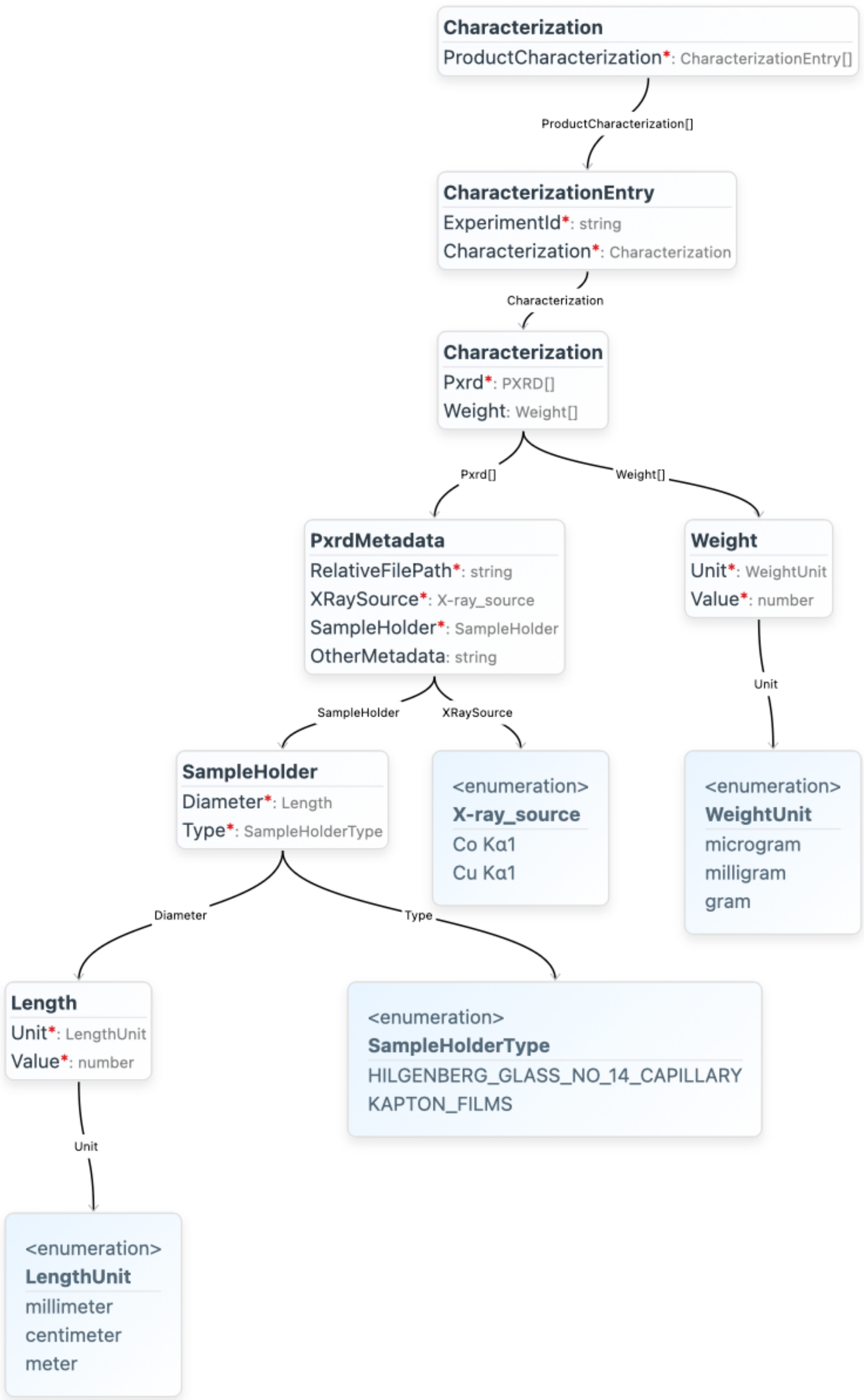

Figure S2. Graph representation of the data model for MOF characterization, drawn by the software MetaConfigurator. Red asterisks denote required properties. The classes are described in more detail in Table S3. Boxes with an enumeration tag denote sets of permitted values.

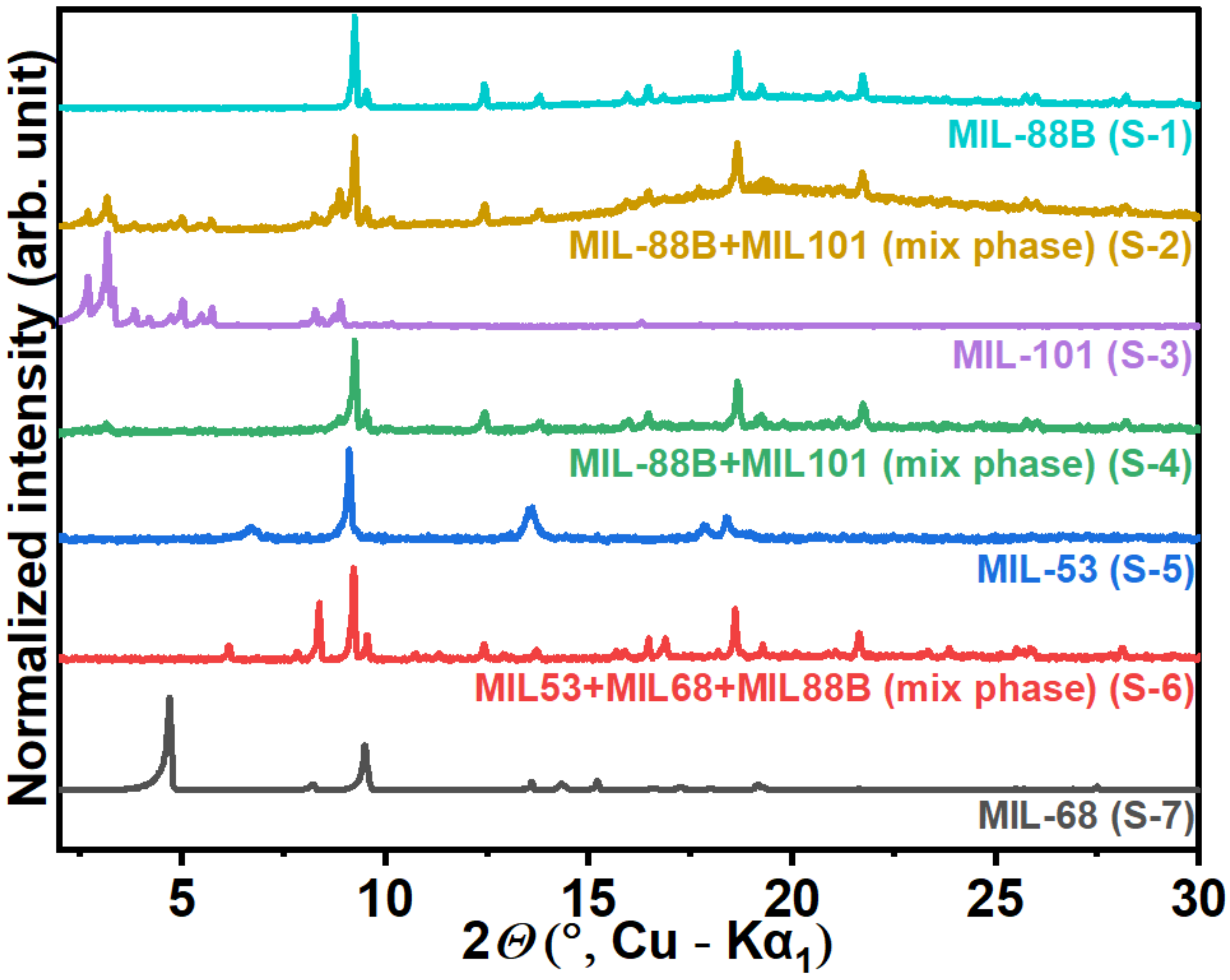

Figure S3. PXRD patterns (Cu Kα_1) of the as-synthesized Fe–terephthalate MOFs.

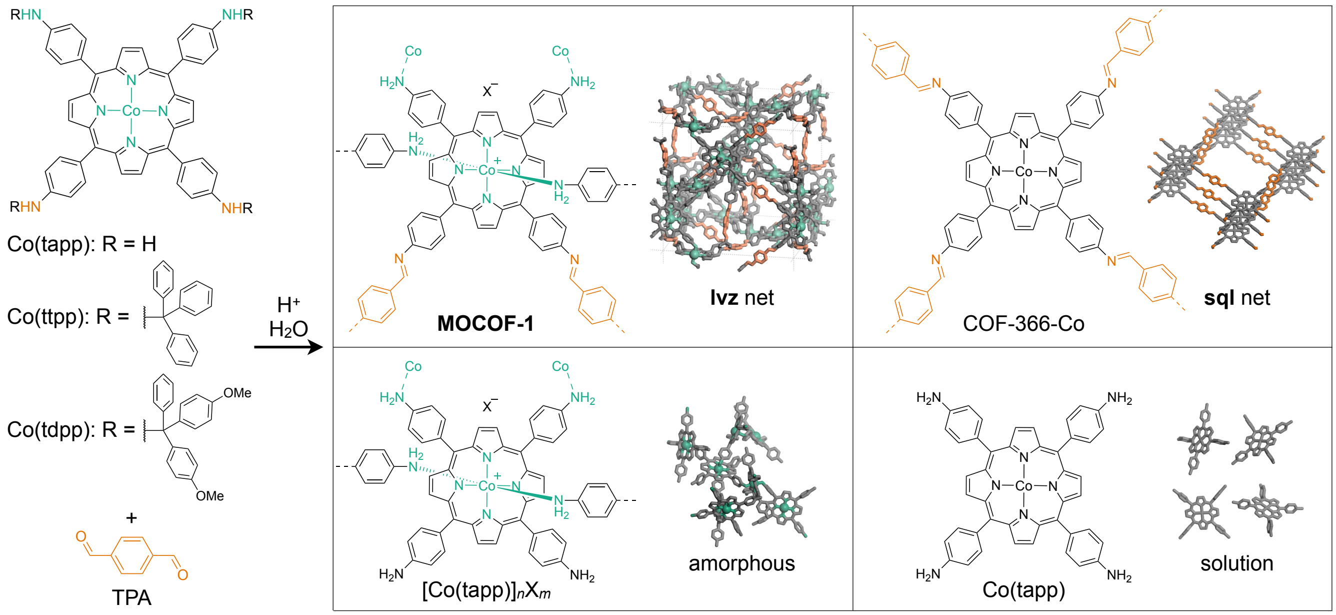

Figure S4. Synthetic scheme and structure of MOCOF-1 and the side phases COF-366-Co, $[Co(tapp)]_nX_m$, and Co(tapp). Metal–organic linkages and covalent organic linkages are colored in green and orange, respectively. The structures of MOCOF-1 and COF-366-Co are determined by single-crystal and powder X-ray diffraction, respectively. The structure of $[Co(tapp)]_nX_m$ is modeled as one possibility.

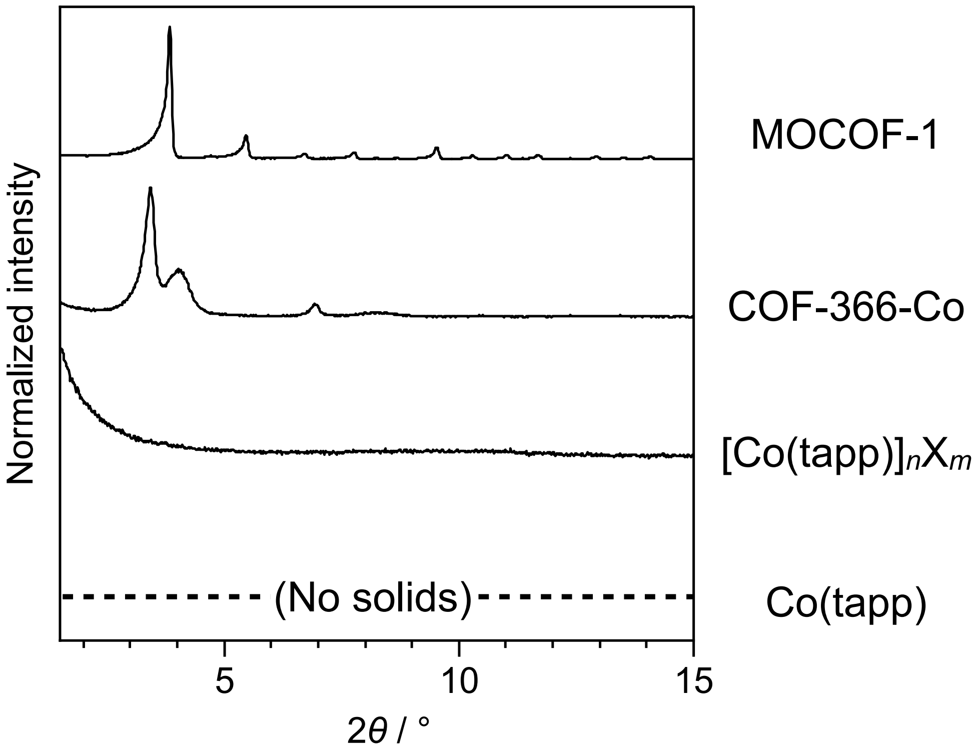

Figure S5. PXRD patterns (Cu Kα_1) of each phase in the MOCOF-1 synthesis.

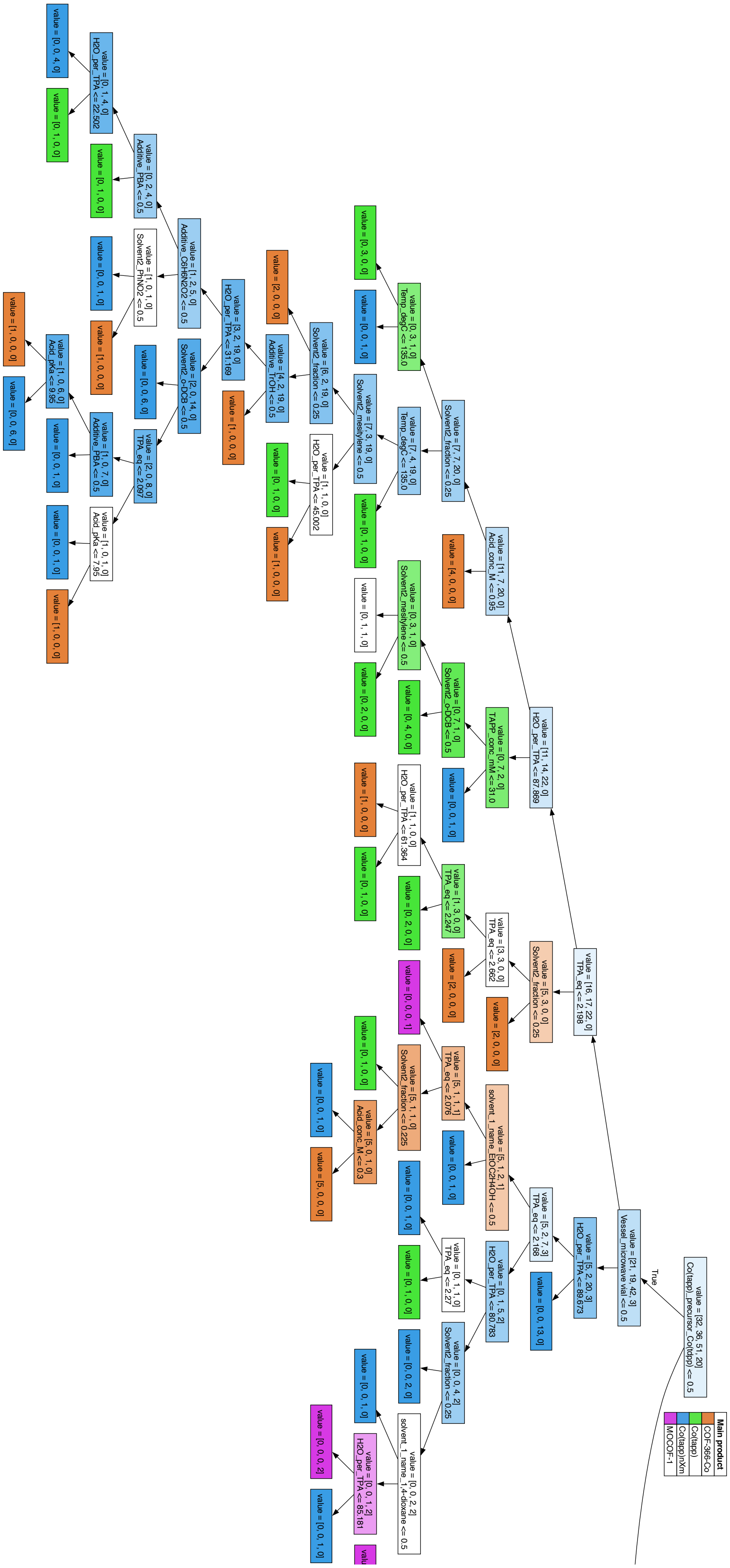
Main product
COF-366-Co
Co(tapp)
Co(tapp)nXm
MOCOF-1
value = [32, 36, 51, 20]
Co(tapp)_precursor_Co(tdpp) <= 0.5
True
value = [21, 19, 42, 3]
Vessel_microwave vial <= 0.5
value = [16, 17, 22, 0]
TPA_eq <= 2.198
value = [11, 14, 22, 0]
H2O_per_TPA <= 87.869
value = [11, 7, 20, 0]
Acid_conc_M <= 0.95
value = [4, 0, 0, 0]
value = [7, 7, 20, 0]
Solvent2_fraction <= 0.25
value = [0, 3, 1, 0]
Temp_degC <= 135.0
value = [0, 3, 0, 0]
value = [0, 0, 1, 0]
value = [7, 4, 19, 0]
Temp_degC <= 135.0
value = [0, 1, 0, 0]
value = [7, 3, 19, 0]
Solvent2_mesitylene <= 0.5
value = [6, 2, 19, 0]
Solvent2_fraction <= 0.25
value = [2, 0, 0, 0]
value = [4, 2, 19, 0]
Additive_TfOH <= 0.5
value = [1, 0, 0, 0]
value = [3, 2, 19, 0]
H2O_per_TPA <= 31.169
value = [1, 2, 5, 0]
Additive_C6H6N2O2 <= 0.5
value = [0, 2, 4, 0]
Additive_PBA <= 0.5
value = [0, 1, 0, 0]
value = [0, 1, 4, 0]
H2O_per_TPA <= 22.502
value = [0, 1, 0, 0]
value = [0, 0, 4, 0]
value = [1, 0, 1, 0]
Solvent2_PhNO2 <= 0.5
value = [0, 0, 1, 0]
value = [1, 0, 0, 0]
value = [2, 0, 14, 0]
Solvent2_o-DCB <= 0.5
value = [0, 0, 6, 0]
value = [2, 0, 8, 0]
TPA_eq <= 2.097
value = [1, 0, 7, 0]
Additive_PBA <= 0.5
value = [1, 0, 6, 0]
Acid_pKa <= 9.95
value = [1, 0, 0, 0]
value = [0, 0, 6, 0]
value = [0, 0, 1, 0]
value = [1, 0, 1, 0]
Acid_pKa <= 7.95
value = [0, 0, 1, 0]
value = [1, 0, 0, 0]
value = [1, 1, 0, 0]
H2O_per_TPA <= 45.002
value = [0, 1, 0, 0]
value = [1, 0, 0, 0]
value = [0, 7, 2, 0]
TAPP_conc_mM <= 31.0
value = [0, 7, 1, 0]
Solvent2_o-DCB <= 0.5
value = [0, 3, 1, 0]
Solvent2_mesitylene <= 0.5
value = [0, 1, 1, 0]
value = [0, 2, 0, 0]
value = [0, 4, 0, 0]
value = [0, 0, 1, 0]
value = [5, 3, 0, 0]
Solvent2_fraction <= 0.25
value = [2, 0, 0, 0]
value = [3, 3, 0, 0]
TPA_eq <= 2.662
value = [2, 0, 0, 0]
value = [1, 3, 0, 0]
TPA_eq <= 2.247
value = [1, 1, 0, 0]
H2O_per_TPA <= 61.364
value = [1, 0, 0, 0]
value = [0, 1, 0, 0]
value = [0, 2, 0, 0]
value = [5, 2, 20, 3]
H2O_per_TPA <= 89.673
value = [0, 0, 13, 0]
value = [5, 2, 7, 3]
TPA_eq <= 2.168
value = [5, 1, 2, 1]
solvent_1_name_EtOC2H4OH <= 0.5
value = [5, 1, 1, 1]
TPA_eq <= 2.076
value = [0, 0, 0, 1]
value = [5, 1, 1, 0]
Solvent2_fraction <= 0.225
value = [0, 1, 0, 0]
value = [5, 0, 1, 0]
Acid_conc_M <= 0.3
value = [0, 0, 1, 0]
value = [5, 0, 0, 0]
value = [0, 0, 1, 0]
value = [0, 1, 5, 2]
H2O_per_TPA <= 80.783
value = [0, 1, 1, 0]
TPA_eq <= 2.27
value = [0, 0, 1, 0]
value = [0, 1, 0, 0]
value = [0, 0, 4, 2]
Solvent2_fraction <= 0.25
value = [0, 0, 2, 0]
value = [0, 0, 2, 2]
solvent_1_name_1,4-dioxane <= 0.5
value = [0, 0, 1, 0]
value = [0, 0, 1, 2]
H2O_per_TPA <= 85.181
value = [0, 0, 0, 2]
value = [0, 0, 1, 0]

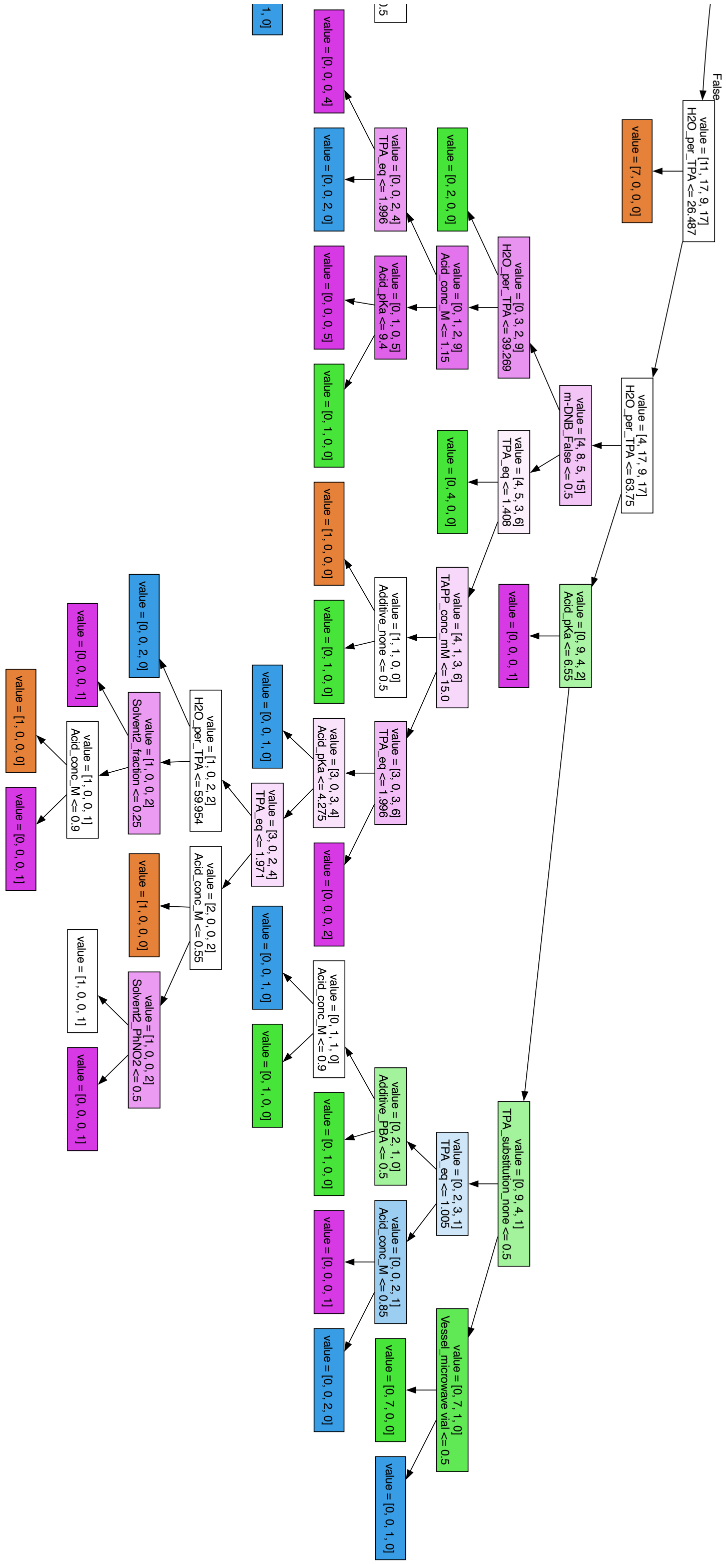

Figure S6. Decision tree visualizing the relationships between the synthesis conditions and the main products. Each node is coloured with its major class with a hue showing its majority.
